# DotU and VgrG, Core Components of Type VI Secretion Systems, Are Essential for *Francisella* LVS Pathogenicity

**DOI:** 10.1371/journal.pone.0034639

**Published:** 2012-04-13

**Authors:** Jeanette E. Bröms, Lena Meyer, Moa Lavander, Pär Larsson, Anders Sjöstedt

**Affiliations:** 1 Clinical Bacteriology, Department of Clinical Microbiology, and Laboratory for Molecular Infection Medicine Sweden (MIMS), Umeå University, Umeå, Sweden; 2 Division of CBRN Defense and Security, Swedish Defense Research Agency, Umeå, Sweden; Université Paris Descartes - INSERM - U1002, France

## Abstract

The Gram-negative bacterium *Francisella tularensis* causes tularemia, a disease which requires bacterial escape from phagosomes of infected macrophages. Once in the cytosol, the bacterium rapidly multiplies, inhibits activation of the inflammasome and ultimately causes death of the host cell. Of importance for these processes is a 33-kb gene cluster, the *Francisella* pathogenicity island (FPI), which is believed to encode a type VI secretion system (T6SS). In this study, we analyzed the role of the FPI-encoded proteins VgrG and DotU, which are conserved components of type VI secretion (T6S) clusters. We demonstrate that in *F. tularensis* LVS, VgrG was shown to form multimers, consistent with its suggested role as a trimeric membrane puncturing device in T6SSs, while the inner membrane protein DotU was shown to stabilize PdpB/IcmF, another T6SS core component. Upon infection of J774 cells, both Δ*vgrG* and Δ*dotU* mutants did not escape from phagosomes, and subsequently, did not multiply or cause cytopathogenicity. They also showed impaired activation of the inflammasome and marked attenuation in the mouse model. Moreover, all of the DotU-dependent functions investigated here required the presence of three residues that are essentially conserved among all DotU homologues. Thus, in agreement with a core function in T6S clusters, VgrG and DotU play key roles for modulation of the intracellular host response as well as for the virulence of *F. tularensis*.

## Introduction

Gram-negative bacteria rely on protein secretion systems (denoted type I to type VII) to mediate successful colonization of hosts [1]. The type VI secretion system (T6SS) was first discovered in *Vibrio cholerae* in 2006 [2], but has since then been identified in more than one fourth of all sequenced bacterial genomes [3], [4], [5]. Many of these T6SS-containing bacteria are known pathogens that rely on T6SSs to mediate infection of eukaryotic hosts (reviewed in [6]), however, type VI secretion (T6S) also play an important role in interbacterial interactions [7].

T6SS gene clusters are suggested to form four or five major phylogenetic groups [3], [4]. Despite large heterogeneity, most systems encode homologues of *V. cholerae* IcmF, DotU, ClpV, VipA, VipB, VgrG and Hcp proteins [4]. IcmF and DotU show homology to proteins from the *Legionella pneumophila* Dot/Icm type IV secretion system (T4SS), where they are thought to interact with each other, thereby stabilizing the secretion machinery [8]. In T6SSs, IcmF is essential for secretion of Hcp in pathogens like *V. cholerae*, *Pseudomonas aeruginosa*, *Aeromonas hydrophila*, *Edvardsiella tarda* and *Agrobacterium tumefaciens* [2], [9], [10], [11], [12], and has been shown to physically interact with DotU [11], [12]. In many species, VipA and VipB proteins have also been shown to interact [13], [14], [15], and in some cases, assemble into tubular structures suggested to span the bacterial membranes [13], [16]. The importance of VipA/B for substrate secretion has been experimentally demonstrated [11], [16], [17], [18].

VgrG (valine-glycine repeat protein G) and Hcp (haemolysin co-regulated protein) are the main substrates secreted by T6SSs. They show structural resemblance to the (gp27)3–(gp5)3 spike complex and tail tubes, respectively, of the cell-puncturing apparatus used by T4 bacteriophages to deliver viral DNA into bacterial target cells (reviewed in [6], [19]). Analogously, Hcp may form a tubule-like hollow structure with a trimeric membrane-puncturing VgrG complex situated at the tip, through which macromolecules may be delivered directly into target cells reviewed in [6], [19]). Apart from the membrane-piercing domains, some evolved VgrGs have C-terminal extensions, *e.g.*, the RtxA actin cross-linking domain of *V. cholerae* VgrG1 and the actin-ADP ribosylating VIP-2 domain of *Aeromonas hydrophila* VgrG1, which upon translocation into eukaryotic target cells cause deleterious effects [20], [21]. Still, these so called evolved VgrGs are in minority, suggesting that the short form of VgrG proteins may be the essential form of the protein due to its ability to puncture cells [19]. Intriguingly, T6SS-independent export of VgrG proteins in *Francisella tularensis* and *P. aeruginosa* was recently reported, suggesting the possibility of cross-talk between T6SSs and other secretion pathways [22], [23], [24].

*F. tularensis* is a Gram-negative intracellular pathogen, which causes the zoonotic disease tularemia in humans and many mammals [25]. The pathogenicity requires multiplication within macrophages [26], which is dependent on bacterial escape from phagosomes into the cytosol [27], [28]. After replication, bacterial egress is thought to occur via the induction of host cell-death [29]. The pathogenesis of tularemia appears to be critically dependent on the ability of *F. tularensis* to modulate the host immune response by a number of mechanisms (reviewed in [30], [31]). Genes necessary for all of these events can be found within the *Francisella* pathogenicity island (FPI), predicted to encode a T6SS (reviewed in [30]). Phylogenetically, however, the *Francisella* FPI appears to be unique from all other T6S clusters described so far [3]. In addition, while FPI genes with limited homology to *icmF* (*pdpB*), *dotU*, *vipA* (*iglA*), *vipB* (*iglB*) and *vgrG* exist, genes encoding obvious ClpV and Hcp homologues are absent [15], [22], [32], although IglC was recently suggested to be structurally homologous to Hcp [33]. Moreover, the *F. tularensis* VgrG homologue is significantly smaller than any hitherto described VgrG and without a C-terminal active domain. There are also conflicting reports about the occurrence of FPI-dependent substrate secretion [22], [23]. Together this raises the question of whether the FPI does indeed encode a true T6SS.

In this study, we analyzed the role of DotU and VgrG in *F. tularensis* LVS as these proteins constitute the core of many T6SSs and therefore are likely to perform essential functions. In agreement with a conserved role in T6S clusters, both VgrG and DotU were found to be essential for *F. tularensis* phagosomal escape, intramacrophage replication, cytopathogenicity, inflammasome activation, and virulence in mice.

## Results

### In *silico* analyses of VgrG and DotU

To gain some insight into the functions of VgrG and DotU in *F. tularensis*, in *silico* analyses were performed. For VgrG, InterProScan [34] and CD-Search [35] failed to reveal any characterized domains. Moreover, only limited homologies to non-*Francisella* VgrG proteins were found using an NCBI blastp search and the non-redundant data base (http://blast.ncbi.nlm.nih.gov/Blast.cgi). According to Phyre2 [36], the highest similarity to *F. tularensis* VgrG is exhibited by the C-terminal domain of the tail-associated lysozyme of the T4 bacteriophage (pdb accession 1K28, confidence 99.8%), whereas HHpred [37] suggests a structural similarity also to the tail spike protein of the bacteriophage P2 (pdb 3AQJ, Prob 84.8, E-value 9), as well as a putative adhesin (pdb 3PET, Prob 88.2, E-value 6.9) present in *Bacteroides fragilis*. Thus, our analysis confirms a previous report [22] suggesting that the VgrG protein likely represents a distant structural homolog of the tail spike protein of some bacteriophages. In the same study, *F. tularensis* VgrG was shown to align to the central part (residues 495–689) of VgrG from *V. cholerae*, which overlaps with parts of the C-terminal region of gp27, and most of the C-terminal domain of the gp5 protein [22]. Using Psipred (http://bioinf.cs.ucl.ac.uk/psipred/), the corresponding regions of *F. tularensis* and *V. cholerae* VgrG were predicted to contain multiple beta-strands. Importantly, extensive beta-sheet interactions of the molecular needle ( = gp5, C-terminal domain) are the driving force for trimerization of the whole cell-puncturing complex of bacteriophage T4 [38].

According to InterProScan and CD-Search, *F. tularensis* DotU is a member of the DUF2077 superfamily of “uncharacterized proteins conserved in bacteria”. Therefore, the Conserved Domain Architecture Retrieval Tool (CDART) [39] was used to look for proteins with domain architectures that contain the DUF2077 superfamily domain. Five different architectures were identified; one group (339 proteins) consists of DotU homologues that carry C-terminal extensions resembling the peptidoglycan binding domain of the OmpA/Pal/MotB family. A second group (592 proteins) includes DotU homologues that are shorter in length and contain no additional domains. The organization of DotU proteins into these two major groups has been reported previously [40], [41]. A third group (14 proteins) contains the DUF2077 domain C-terminally fused to a SPOR domain, which is a region found in proteins involved in sporulation and cell division, *e.g.* FtsN, DedD, and CwlM. Similar to the OmpA motif, SPOR is involved in binding to peptidoglycan [42]. Finally, in a fourth (9 proteins) and fifth group (3 proteins) the DUF2077 domain is N-terminally fused to a Motile_sperm domain (Eurotiomycetes) or SRPBCC domain (β-proteobacteria), respectively. The existence of these different variants suggests that the DUF2077 superfamily and DotU may have adapted to perform distinct functions in different organisms. A phylogenetic analysis of DUF2077, including sequences from group 1 to 3, demonstrated significant diversity and large protein distances across the tree (Figure S1). Reliable bipartitions could only be obtained for related homologues. The bootstrap support was very low for deep branch points within the tree, demonstrating the uncertainty of phylogenetic relationships at greater distances. The multi-domain proteins containing the DUF2077 and OmpA domains, and DUF2077 and SPOR domains, respectively, were found to form genetic clusters (Figure S1). Within the clusters, however, at a low frequency, also single-domain DotU homologues could be found, which may indicate that the second domain occasionally has been lost during the evolution of these homologues. The DotU homologues found to be most closely related to DotU of *F. tularensis* were those present in *Stigmatella aurantica*, *Desulfonatronospira thiodismutans* and *Haliangium ochraceum* (Figure S1). This was confirmed by performing an NCBI protein blast using the Non-redundant protein database. Here, DotU from *D. thiodismutans* was found to be the closest homologue to *F. tularensis* DotU (e-value 0.001; 32% identity) followed by the DotU protein of *H. ochraceum* (e-value 0.021; 23% identity).

Intriguingly, multiple sequence alignments using MSAprobs v. 0.9.5 [47] revealed three residues that were conserved in almost all proteins of the DUF2077 superfamily (Figure S2). These corresponded to Asp70, Glu71 and Gly134 of *F. tularensis* DotU. The aspartic acid residue was conserved in all but one protein (asparagine) of the 653 homologues. The glutamic acid residue was also highly conserved, but was exchanged for aspartic acid in 24 of the homologues or for other amino acids in four homologues. The glycine residue was occasionally exchanged for glutamine (6), serine (2), alanine (1), histidine (1) and valine (1). Despite a high conservation at these residues, however, the overall conservation of the DotU homologues was low. Only 55 amino acid residues could be properly aligned across all proteins (data not shown). Using Psipred to predict secondary structures, DotU proteins are predominantly α-helical proteins with the conserved Asp and Glu residues residing within an α-helix and the conserved Gly located in between two helices (data not shown). Using Phyre2 or HHpred with *F. tularensis* DotU as a query, no significant hits could be predicted by either method, demonstrating the dissimilarity of DotU to any protein of known structure.

### Construction of Δ*vgrG* and Δ*dotU* null mutants

To determine the role of VgrG and DotU in *F. tularensis* LVS, in-frame deletion mutants were constructed, by deletion of both copies of each gene. The resulting null mutant strains Δ*vgrG* and Δ*dotU* were used in various biological assays. To verify the absence of VgrG expression in the Δ*vgrG* mutant, immunoblot analysis with anti-VgrG antibodies was performed on bacterial pellets (data not shown). We are, however, currently lacking antibodies against DotU. Therefore, real-time PCR was used to quantify levels of *vgrG* and *dotU* transcripts in the Δ*vgrG* and Δ*dotU* mutants, respectively. In both cases, levels were below the detection limit of the assay (data not shown). For complementation in *trans*, *vgrG* and *dotU* were expressed from the *groE* promoter of pMOL52, a derivative of pKK289Km expressing GSK (Glycogen synthase kinase) [23], in the Δ*vgrG* and Δ*dotU* mutants, respectively. This resulted in strains Δ*vgrG*/pVgrG_GSK_ (pMOL54) and Δ*dotU*/pDotU_GSK_ (pMOL58). Importantly, tagged versions of VgrG and DotU behaved identical to their non-tagged counterpart expressed from the same promoter in all subsequent analyses (data not shown), suggesting that the tag is not likely to impact on the protein functions.

### VgrG and DotU are essential for phagosomal escape and intramacrophage growth

**Figure 1:**
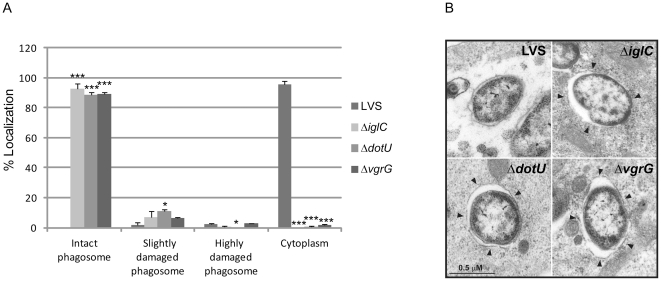
Phagosomal escape of *F. tularensis*. J774 cells were infected with *F. tularensis* at a MOI of 1000 for 2 h and, after washing incubated for another 6 h before they were fixed and analyzed by transmission electron microscopy (TEM). (A) Bacteria were divided into one of four categories based on the membrane integrity of the surrounding vacuolar membrane. Each bar represents the mean values and the error bar indicates the standard deviation from two different sections. Asterisks denote that the % of localization is statistically different from LVS as determined by a 2-sided *t*-test with equal variance (*, *P*≤0.05; ***, *P*≤0.001). (B) Electron micrographs of infected J774 cells were acquired with a JEOL JEM 1230 Transmission Electron Microscope (JEOL Ltd., Tokyo, Japan). Black arrows indicate vacuolar membranes surrounding intracellular bacteria. Scale bar 0.5 µm.

Many FPI mutants have been shown to be defective for the escape from the phagosomal compartment (reviewed in [30]). To determine whether *F. tularensis* has an intraphagosomal localization, the most frequently used marker is LAMP-1, which is a late endosomal and lysosomal marker acquired within 30 min by the *Francisella*-containing phagosome (reviewed in [31]). Thus, confocal microscopy was used to determine the percentage of LAMP-1 colocalization for Δ*vgrG* or Δ*dotU* mutant bacteria expressing Green fluorescent protein (GFP) 3 h post infection in J774 macrophages. At this time point, only 6.8±3.3% of LVS, the positive control strain, colocalized with LAMP-1, while the corresponding numbers for Δ*iglC*, the negative control, were 92.0±3.2% (*P*<0.001 vs. LVS). For the Δ*vgrG* and Δ*dotU* mutants, the numbers were 92.8±3.0% and 83.2±4.8% respectively (both *P*<0.001 vs. LVS), suggesting that these mutants, similar to Δ*iglC*, do not escape from the phagosomes. To corroborate the results from the confocal microscopy, also transmission electron microscopy was performed. J774 cells were infected with LVS, Δ*vgrG*, Δ*dotU* or Δ*iglC* mutants and the percentage of bacterial escape 6 h post infection determined. This is a relevant time point to distinguish between LVS mutants that show no or delayed escape [23]. At 6 h, the majority of LVS bacteria were found free in the cytoplasm (95.5±2.1%), while a small population was surrounded by highly damaged (<50% of membrane intact) vacuolar membranes (2.5±1.4%) (Figure 1A and B). At the same time point, a majority of Δ*vgrG* (89.0±2.1%), Δ*dotU* (88.5±1.4%), and Δ*iglC* (92.5±2.5%) mutant bacteria was surrounded by intact vacuolar membranes (all *P*<0.001 vs. LVS) or slightly damaged vacuolar membranes (>50% of membrane intact) (6.5±0.7%, 11.0±1.4%, and 7.0±4.2%, respectively) (Figure 1A and B). This suggests that Δ*vgrG* and Δ*dotU*, similar to Δ*iglC*, do not escape from the phagosome in analogy to what we observed in the LAMP-1 colocalization experiment. Similar findings have also been shown for a *F. novicida vgrG* mutant [22].

**Figure 2:**
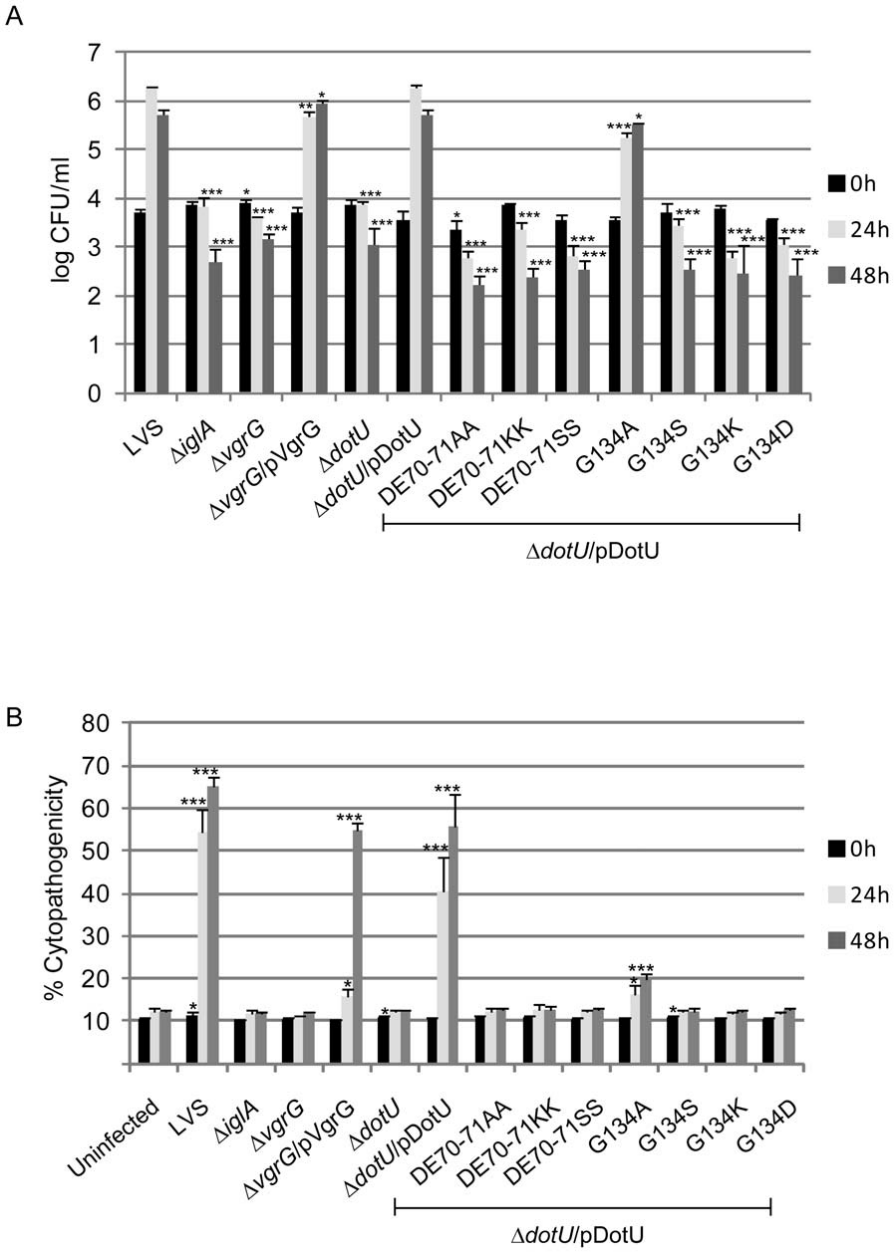
Intracellular growth (A) and cytopathogenicity (B) of *F. tularensis* strains. (A) J774 cells were infected by various strains of *F. tularensis* at an MOI of 200 for 2 h. Upon gentamicin treatment, cells were allowed to recover for 30 min after which they were lysed immediately (corresponds to 0 h; black bars) or after 24 h (light gray bars) or 48 h (dark gray bars) with PBS-buffered 0.1% sodium deoxycholate solution and plated to determine the number of viable bacteria (log_10_). All infections were repeated three times and a representative experiment is shown. Each bar represents the mean values and the error bar indicates the standard deviation from triplicate data sets. The asterisks indicate that the log_10_ number of CFU was significantly different from the parental LVS strain as determined by a 2-sided *t*-test with equal variance (*, *P*≤0.05; **, *P*≤0.01; ***, *P*≤0.001). (B) Culture supernatants of infected J774 cells were assayed for LDH activity at 0, 24 and 48 h post infection and the activity was expressed as a percentage of the level of non-infected lysed cells (positive lysis control). Shown are means and standard deviations of triplicate wells from one representative experiment of two. The asterisks indicate that the cytopathogenicity levels were significantly higher than those of uninfected cells at a given time point as determined by a 2-sided *t*-test with equal variance (*, *P*≤0.05; ***, *P*≤0.001).

Mutants previously shown to be impaired for phagosomal escape, have also been defective for intracellular growth (reviewed in [30]). Therefore, viable counts were performed at different time points post infection, to determine the ability of Δ*vgrG* and Δ*dotU* mutants to multiply within the host cytosol. Similar to the negative control strain Δ*iglA* [14], neither mutant showed any substantial growth over a time period of 48 h (Figure 2A), which corroborates the recent findings for Δ*vgrG* and Δ*dotU* mutant strains of *F. novicida* [33]. The Δ*dotU* mutant complemented in *trans* behaved similarly to LVS at all time points tested. In contrast, while the complemented Δ*vgrG* mutant exhibited efficient growth, it was slightly delayed at 24 h (*P*<0.01 vs LVS), but exceeded that of LVS at 48 h (*P*<0.05) (Figure 2A). Partial complementation for growth was also observed for a *vgrG* mutant of *F. novicida* when complemented in *trans* [22]. Most likely, this phenotype is a direct consequence of the altered stoichiometry caused by the constitutive and/or high expression of *vgrG* in *trans*. In support, complementation of Δ*vgrG* in *cis* (Δ*vgrG*/VgrGcis) efficiently restored the ability of the mutant to grow within J774 cells (102% of LVS at 24 h, *P* = 0.21 and 100% of LVS at 48 h; *P* = 0.75).

Importantly, neither Δ*vgrG* nor Δ*dotU* showed any growth defect in *vitro* when cultivated in Chamberlain's defined medium over a time period of 24 h (data not shown). Thus, VgrG and DotU are essential for phagosomal escape and subsequently intramacrophage multiplication.

### Δ*vgrG* and Δ*dotU* mutants do not induce a cytopathogenic response

Defects in phagosomal escape and cytosolic replication generally correlate with a lack of cytopathogenicity (reviewed in [30]). The cytopathogenic response induced by LVS is characterized by morphological changes such as membrane blebbing, cell detachment, and LDH release [29], [48]. To determine whether Δ*vgrG* and Δ*dotU* are able to induce cytopathogenicity, J774 cells were infected with the null mutant strains and the release of LDH into the cell culture supernatants measured. In addition, the morphological effect on cells was investigated using phase contrast microscopy. At 24 h post infection, LVS induced significant LDH release from the infected cells, which also were notably morphologically affected (Figure 2B and data not shown). At 24 and 48 h post infection, LDH levels in culture supernatants and the morphological appearance of cells infected with either of Δ*vgrG* or Δ*dotU* did not differ much from that of cells infected with the negative control strain Δ*iglA* or uninfected cells (Figure 2B and data not shown). Expression of DotU in *trans* efficiently restored the cytopathogenic response of the Δ*dotU* mutant (75% of LVS at 24 h, *P* = 0.07 and 85% of LVS at 48 h, *P* = 0.10) (Figure 2B). Although delayed at 24 h (29% of LVS, *P*<0.001), the Δ*vgrG* mutant complemented in *trans* also induced an efficient cytopathogenic response at 48 h (84% of LVS, *P*<0.01), while the Δ*vgrG* mutant complemented in *cis*, behaved indistinguishable from LVS (95% of LVS at 24 h, *P* = 0.5 and 99% of LVS at 48 h, *P* = 0.9). Thus, VgrG and DotU are clearly essential for the ability of *Francisella* LVS to induce prominent cytopathogenicity.

### The requirement of VgrG and DotU for modulation of macrophage inflammatory responses

**Figure 3:**
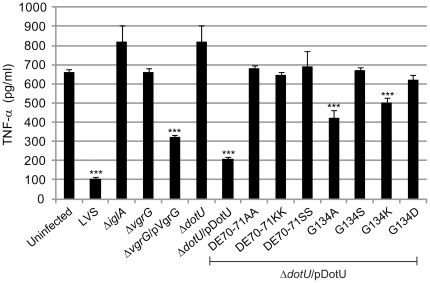
TNF-α secretion of *F. tularensis* infected J774 cells. J774 cells left uninfected (-) or infected with *F. tularensis* at an MOI of 500 for 2 h, were washed and subsequently incubated in the presence of *E. coli*-derived LPS (50 ng/ml) for an additional 2 h. The average TNF-α secretion in pg/ml and standard errors from two experiments, in which quintuplicate samples (n = 5) were used is shown. In the absence of LPS, the cytokine levels were below the limit of detection for the assay (<15 pg/ml) (data not shown). A Student's 2-sided *t*-test was used to determine whether the TNF-α secretion induced by the various *F. tularensis* strains was significantly different from that of the uninfected control (***, *P*<0.001).

Pro-inflammatory cytokines, such as TNF-α, are critical mediators of an effective defense against *Francisella* infection. In *vitro* studies using mouse macrophages and human monocytes demonstrated that *F. tularensis* actively suppressed the ability of host cells to produce and secrete TNF-α in response to *E. coli* LPS, an inflammasome-independent process [49], [50]. *F. tularensis*-mediated suppression of cytokine production also occurred following in *vivo* pulmonary infection and also has been shown to occur in human dendritic cells [51], [52]. Mutants within *iglA*, *iglC*, *iglG* or *iglI* do not inhibit suppression of TNF-α secretion upon infection, suggesting that the FPI is crucial for this event [23], [49]. To characterize the contribution of VgrG and DotU to suppression, J774 cells were infected with Δ*vgrG* or Δ*dotU* as well as the complemented mutant strains using a high MOI (500) to ensure that 100% of the cells become infected [49]. After 2 h of stimulation with *E. coli* LPS, cell culture supernatants were collected and assayed for the presence of TNF-α. Efficient inhibition of TNF-α production was observed for LVS (Figure 3). In contrast, the control strain Δ*iglA* did not inhibit TNF-α release, similar to Δ*vgrG*, Δ*dotU* and the uninfected control (Figure 3). Inhibition could, however, be partially restored by expressing VgrG in *trans* in Δ*vgrG* and DotU in *trans* in Δ*dotU* (Figure 3). Thus, VgrG and DotU are required for efficient inhibition of TNF-α production in infected macrophages.

Phagosomal escape of *F. tularensis* into the macrophage cytosol is critical for the inflammasome-dependent induction of IL-1β secretion [22], [23], [53], [54], [55], [56]. As a result, macrophages infected with mutants in *iglC*, *iglG*, *iglI* or *vgrG* exhibit diminished IL-1β release [22], [23], [53], [55], [57]. To confirm the previous data on the importance of VgrG for IL-1β release and to determine whether also DotU play a role in this process, the concentration of IL-1β was measured in culture supernatants of macrophages infected with the corresponding LVS mutants at 5 or 24 h post infection. Mouse peritoneal exudate cells (PECs) infected with LVS induced high levels of IL-1β release (Table 1). In contrast, PEC cells infected with Δ*vgrG*, Δ*dotU*, the negative control strain Δ*iglA*, or uninfected cells produced levels of IL-1β secretion that were below detection levels of the assay (<31.25 pg/ml) both at 5 and 24 h (Table 1). Upon complementation in *trans*, IL-1β secretion was partially restored in Δ*vgrG* as well as in Δ*dotU* (Table 1). Thus, VgrG and DotU are both essential for the IL-1β release by LVS, consistent with their inability to escape from the phagosome. These data support the notion of a strong correlation between inflammasome activation and a cytoplasmic location of the bacterium [22], [23], [53], [55], [57].

**Table 1:** IL-1β secretion of *F. tularensis* infected PEC cells.

	IL-1β secretion (pg/ml)^a^	
Strain	5 h	24 h
*-*	BDL	BDL
LVS	94.3±16.4	394.6±60.5
Δ*iglA*	BDL	BDL
Δ*vgrG*	BDL	BDL
Δ*vgrG*/pVgrG	68.0±18.5	102.6±9.8***
Δ*dotU*	BDL	BDL
Δ*dotU*/pDotU	56.3±7.1*	270.9±41.7

a *Francisella*-infected (MOI = 200) or non-infected (-) PEC cells were incubated for 5 or 24 h after gentamicin treatment. The average IL-1β secretion in pg/ml and standard errors from triplicate samples (n = 3) from one experiment is shown. BDL means that the cytokine levels were below the limit of detection for the assay (<31.25 pg/ml). A Student's 2-sided *t*-test was used to determine whether the IL-1β release induced by the complemented mutants were significantly different to the parental strain (*, *P*<0.05; ***, *P*<0.001).

### VgrG and DotU are required for virulence

The strongly attenuated phenotypes observed for the Δ*vgrG* and Δ*dotU* null mutants with respect to phagosomal escape, intracellular growth, LDH release and an inflammatory response suggested that they are likely to be attenuated in *vivo*. To test this, C57BL/6 mice were infected by the intradermal route. With an infection dose of 4×10^7^ CFU (approximately 2×LD_50_) [58], LVS caused 80% mortality (mean time to death 4.3±0.5 days). In contrast, no mice died after infection with ∼6×10^8^ CFU of Δ*vgrG* or Δ*dotU*. While complementation of Δ*dotU* resulted in 20% mortality with a dose of ∼4×10^7^ CFU, and 80% mortality with a dose of ∼7.5×10^8^ CFU (mean time to death 4.8±0.5 days), the killing capacity of Δ*vgrG* could not be restored upon complementation in *trans* (dose ∼5×10^8^ CFU) (Table 2). Perhaps this lack of complementation can be explained by the delay in intramacrophage growth and cytopathogenicity exhibited by this strain (above). Regardless, these results suggest that VgrG and DotU are important virulence determinants of LVS. To determine the bacterial burden in organs, spleens were isolated on day 12 p.i. At this time point, Δ*vgrG* had been essentially cleared, while some Δ*dotU* mutant bacteria could still be recovered from slightly enlarged spleens (Table 2). Still, the Δ*dotU* infected animals showed no other obvious signs of infection.

**Table 2:** Intradermal infections of mice.

Strain	Dose	Surviving mice (MTD)	CFU/ml in spleen
LVS	4×10^7^	1/5 (4.3±0.5 days)	NT
LVS	5×10^7^	1/5 (3.5±0.6 days)	10
Δ*vgrG*	6×10^8^	5/5	30; 0; 0; 0; 0
Δ*vgrG/*pVgrG	5×10^8^	5/5	0; 0; 0; 0; 0
Δ*dotU*	6×10^8^	5/5	360; 240; 280; 10; 2380
Δ*dotU*	1×10^9^	5/5	50, 0; 0; 10; 60
Δ*dotU*/pDotU	4×10^7^	4/5 (4.0±0.0 days)	0; 0; 0; 0
Δ*dotU*/pDotU	7.5×10^8^	1/5 (4.8±0.5 days)	0
Δ*dotU*/pDotU	1×10^9^	0/5 (4.0±0.0 days)	
Δ*dotU*/pDotU _G134A_	1×10^9^	4/5 (4.0±0.0 days)	0; 0; 0; 0; 0
Δ*dotU*/pDotU _DE70-71SS_	1×10^9^	5/5	170; 0; 0; 0; 0
Δ*dotU*/pDotU _G134S_	1×10^9^	5/5	1330; 0; 0; 370; 1110

C57BL/6 female mice (n = 5) were infected intradermally with different strains of *F. tularensis* in 2 representative experiments. The doses given are the approximate numbers of *F. tularensis* injected (for real doses see Materials and Methods). The number of surviving mice as well as the mean time to death (MTD) and standard deviations are shown. When indicated, spleens were isolated on day 12 p.i and the numbers of CFU/ml determined. NT = not tested.

### Membrane integrity of Δ*vgrG* and Δ*dotU* mutants

Because of their dramatic phenotypes (above) and since DotU has been shown to localize to the bacterial inner membrane in some bacteria [8], [59], we considered that Δ*dotU* and Δ*vgrG* may be defective for membrane integrity and/or sensitive to stress stimuli. The LPS profiles from the mutants were, however, indistinguishable from those of LVS (data not shown). Moreover, none of the mutants show increased susceptibility to detergents (SDS), dye (EtBr) or antibiotics (Vancomycin) [54], nor to stress-related stimuli like low pH or H_2_O_2_ (data not shown). Therefore, the membrane integrity of the mutants appeared to be intact.

### VgrG is capable of multimerization

**Figure 4:**
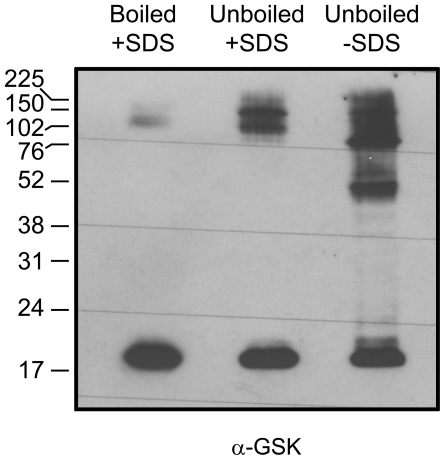
Multimeric complexes formed by VgrG in *F. tularensis* LVS. VgrG proteins contained in the pellet fraction of a Δ*vgrG* null mutant expressing VgrG-GSK in *trans* were separated by SDS-PAGE and identified by immunoblot using antiserum specific to GSK. Large, multimeric VgrG complexes are formed when SDS and/or sample boiling are omitted. Amersham full range rainbow molecular weight marker was used in this analysis and the sizes in kDa are indicated. The experiment was repeated at least two times and a representative example is shown.

**Figure 5:**
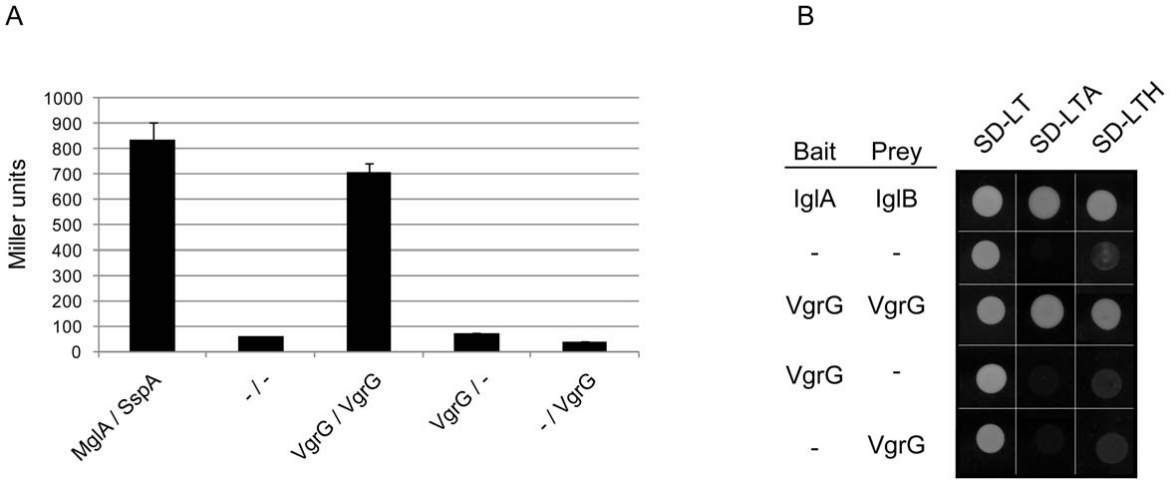
VgrG forms homodimers. (A) Bacterial 2-Hybrid analysis of VgrG-VgrG interactions. Contact between VgrG proteins fused to Zif and to the ω subunit of *E. coli* RNAP, induces transcription from the *lacZ* promoter of the *E. coli* reporter strain KDZif1ΔZ, resulting in β-gal activity. The negative control corresponds to vectors without insert, while the positive control is MglA-SspA [61]. Shown is the mean β-gal activity ± standard deviation in Miller units produced from three independent experiments, where two independent transformants were tested on each occasion. (B) Yeast Two-hybrid analysis of VgrG-VgrG interactions. VgrG fused to the GAL4 activation domain of plasmid pGADT7 or the GAL4 DNA-binding domain pGBKT7 were co-transformed into the *S. cerevisiae* reporter strain AH109. VgrG dimer formation results in activation of the *ADE2* and *HIS3* reporter genes, to allow growth of yeast on minimal SD medium devoid of adenine (-LTA) and histidine (-LTH) respectively at 30°C. The negative control corresponds to vectors without insert, while the positive control is IglA-IglB [14]. Results reflect trends in growth from three independent experiments in which several individual transformants were tested on each occasion.

Previously, VgrG overproduction in *P. aeruginosa* has been shown to result in the occurrence of a large number of high molecular weight bands in the supernatants [24], [60]. Furthermore, when the samples were not boiled to reduce disruption of protein complexes, the amounts of VgrG complexes were significantly increased [24]. Thus, VgrG of *P. aeruginosa* evidently interacts with itself. Furthermore, the *V. cholerae* VgrG1, VgrG2 and VgrG3 proteins have been shown to interact in various combinations [20]. To test whether *F. tularensis* VgrG can interact with itself, bacterial pellets were analyzed for the presence of VgrG multimers using either boiled or unboiled samples prepared in sample buffer with or without SDS. When expressed from its native promoter on the chromosome, VgrG levels were very low, and no obvious VgrG multimers were detected within the pellet fractions of LVS (data not shown). In contrast, when VgrG was expressed from pMOL54 in Δ*vgrG*, high molecular weight bands were observed that increased in numbers when the samples had not been boiled, and even more so if SDS was also omitted (Figure 4). This clearly suggested that *F. tularensis* VgrG is capable of multimerization. This was not an artifact caused by the GSK-tag, since GSK-tagged versions of FPI encoded DotU, IglA, IglB, IglC and IglD expressed in the isogenic mutant background did not form multimers when prepared the same way (data not shown). To confirm the occurrence of VgrG-VgrG interactions in *F. tularensis*, two independent protein-protein interaction assays were utilized. First, a Bacterial-2-hybrid (B2H) system was used [61]. *vgrG* was cloned into expression vectors pACTR-AP-Zif and pBRGPω, which resulted in C-terminal VgrG fusions to the Zinc finger DNA-binding domain of murine Zif268 (aa 327–421) and the ω subunit of *E. coli* RNAP respectively. When co-transformed into the *E. coli* reporter strain KDZif1ΔZ, β-gal activity of levels similar to that observed for the positive control (MglA-SspA) [61] was observed (Figure 5A). In contrast, similar to the vector control strain (empty vectors), no β-gal activity was observed when one of the VgrG expression vectors was co-transformed with a empty vector (Figure 5A). This suggests that VgrG is capable of forming homo dimers. This was also confirmed using a Yeast-2-hybrid (Y2H) system. *vgrG* was expressed from the GAL4 activation domain plasmid pGADT7 (prey) and the GAL4 DNA-binding domain plasmid pGBKT7 (bait). When either plasmid was transformed into the reporter yeast strain *Saccharomyces cerevisiae* AH109, it did not result in *ADE2* or *HIS3* reporter gene activation. In contrast, when the plasmids were co-transformed, the *ADE2* and *HIS3* reporter genes were activated to allow growth of yeast on minimal media devoid of adenine and histidine respectively, similar to the positive control strain (IglA-IglB) [14] (Figure 5B).

Based on the structural resemblance to phage tail-spike organelles, Hcp may assemble into a tubular structure, capped by the VgrG trimer and sheathed by the VipA-VipB tubule [19], [62], [63]. Therefore, the possibility that VgrG could interact with IglC (Hcp) [33], IglA (VipA) or IglB (VipB) was tested using the B2H and Y2H systems, however, no interactions could be found (data not shown).

### DotU is essential for IcmF stability

In the *Legionella* T4SS, DotU and IcmF function as accessory factors that stabilize the secretion apparatus [8], [59], rendering them important for efficient *Legionella* intracellular multiplication [8], [64], [65]. The function may involve a DotU-IcmF protein complex, as the former becomes degraded in the absence of the latter [8]. Moreover, in T6SSs, the DotU homologues ImpK_L_ of *A. tumefaciens* and EvpN of *E. tarda* have been shown to physically interact with the IcmF proteins ImpL_M_ and EvpO, respectively [11], [12].

**Figure 6:**
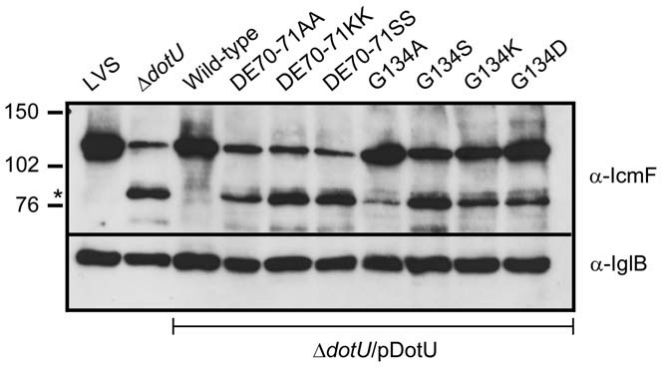
DotU impacts on IcmF/PdpB stability. Analysis of IcmF stability in a Δ*dotU* null mutant expressing GSK-tagged DotU and mutated variants thereof in *trans*. Proteins contained in inner membrane fractions were separated by SDS-PAGE and identified by immunoblot analysis using antiserum specific for IcmF/PdpB or the loading control IglB. The asterisks indicate IcmF degradation products that are formed in the absence of DotU or in the presence of specific DotU substitution mutants expressed in *trans*, and the corresponding sizes in kDa are indicated. Full-length IcmF is predicted to be a 127.5 kDa protein according to SAPS (www.ebi.ac.uk/Tools/saps/). The experiment was repeated at least two times and a representative example is shown.

To determine whether the *Francisella* DotU protein plays a role in stabilizing the putative T6SS of *F. tularensis*, Western blot analysis was used to estimate the levels of various FPI proteins. The recent observation that IcmF/PdpB becomes unstable in a Δ*dotU* mutant of *F. novicida* [33] was extended to also include LVS. Thus, while the anti-IcmF antibody recognized a ∼127.5 kDa protein in the inner membrane fraction of wild-type bacteria, consistent with the predicted molecular mass of IcmF, the homologue in the Δ*dotU* mutant appeared to be partially processed and a smaller form was visible (Figure 6). The reduction in full-length IcmF was not due to any defect in *icmF* transcription (data not shown) and could readily be complemented by expressing DotU in *trans* (Figure 6). Thus, DotU impacts on IcmF stability. No processing/degradation of IglH, IglD, IglC, IglB, IglA, VgrG, or PdpA in the Δ*dotU* mutant was observed (Figure 6 and data not show), suggesting that the instability may be specific to IcmF. To determine whether the loss of stable IcmF in the Δ*dotU* mutant coincides with an ability of DotU to bind to IcmF, the B2H assay was used, however, no production of β-gal activity indicative of an interaction was found (data not shown). This is similar to the finding by Zusman *et al* who also failed to demonstrate an interaction between the *Legionella* homologues using a B2H approach [65]. Since the Y2H system has been successfully used to study DotU-IcmF interactions [11], [12], also this method was applied, however, no interaction between *F. tularensis* DotU and IcmF (full-length IcmF or soluble domain only) could be found using this system either (data not shown).

### Conserved residues within a central domain are crucial for DotU function

Multiple sequence alignments of DotU proteins revealed a striking conservation of three residues, corresponding to Asp70, Glu71 and Gly134 of *F. tularensis* DotU (Figure S2). Therefore, site-directed mutagenesis was performed to analyze their importance for DotU function. To determine if charge and polarity play a role, both of Asp and Glu (polar, negative) were exchanged to Ala (non-polar, neutral), Lys (polar, positive) or Ser (polar, neutral). For the same reason, Gly (non-polar, neutral) in position 134 was exchanged to Ala (non-polar, neutral), Ser (polar, neutral), Lys (polar, positive) or Asp (polar, negative). GSK-tagged versions of the mutated alleles were expressed in *trans* from pKK289Km, and their ability to complement the Δ*dotU* mutant analyzed with respect to intramacrophage growth, cytopathogenicity, TNF-α secretion, IcmF stability and virulence in mice.

**Figure 7:**
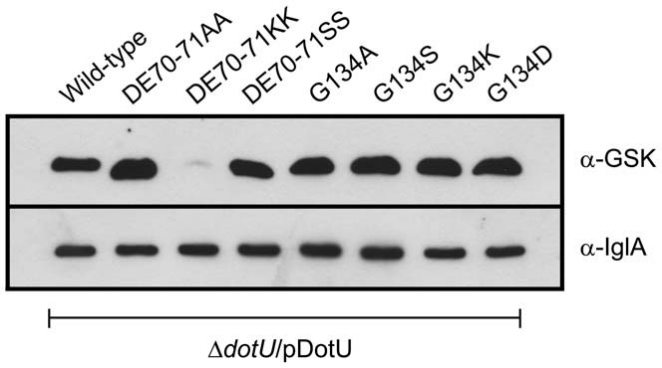
DotU expression in *F. tularensis* LVS. Analysis of DotU protein synthesis from Δ*dotU* expressing GSK-tagged DotU or mutated variants thereof in *trans*. DotU proteins contained in the pellet fraction were separated by SDS-PAGE and identified by immunoblot using an anti-GSK antiserum. Antibodies specific for IglA was used as a loading control. The experiment was repeated two times and a representative example is shown.

Intriguingly, in contrast to wild-type DotU, six out of the seven substitution mutants generated (*i.e.* mutants DE70-71AA, DE70-71KK, DE70-71SS, G134S, G134K and G134D) did not restore intramacrophage growth and cytopathogenicity of Δ*dotU* (Figure 2A and B). Moreover, these mutants also failed to inhibit LPS-induced TNF-α secretion (Figure 3) (only partial suppression observed for the G134K mutant) and were not able to fully stabilize IcmF (Figure 6). These dramatic phenotypes did not correlate with a general defect in protein stability, as only one of the substitutions (DE70-71KK) resulted in a DotU variant that was less abundant in the bacterium (Figure 7). In contrast to the six mutants mentioned above, a seventh mutant was generated that did not display a null mutant phenotype, *i.e.* G134A. Compared to Δ*dotU* expressing wild-type DotU in *trans*, this mutant reached slightly lower numbers in J774 cells at 24 h (83.6% of wild-type DotU, *P*<0.001), while at 48 h the two strains were indistinguishable (Figure 2A). Still, the G134A mutant caused very modest cytopathogenicity (Figure 2B) and only partial inhibition of LPS-induced TNF-α secretion (Figure 3), whereas its ability to stabilize IcmF was more or less preserved (Figure 6). With an infection dose of ∼1×10^9^ CFU, 100% of the mice infected with Δ*dotU* expressing wild-type DotU in *trans* died (mean time to death 4.0±0.0 days), while only 20% of the mice died after receiving the G134A mutant (mean time to death 4.0±0.0 days) and no deaths occurred upon infection with Δ*dotU* or Δ*dotU* expressing either of DE70-71SS or G134S (Table 2). Importantly, this dose was at least 20 times higher than the LD_50_, since infection with ∼5×10^7^ CFU of LVS killed 80% of the mice (mean time to death 3.5±0.6 days). Despite the high dose, very few bacteria could be recovered from the spleens of surviving mice infected by Δ*dotU* or any of the substitution mutants (G134S being the only exception) (Table 2).

Taken together, these results clearly suggest that residues D70 and/or E71, and to a significant extent also G134, all are crucial for the different functions of the DotU protein, and any alteration to their charge or polarity renders *F. tularensis* essentially avirulent.

## Discussion

Intracellular growth is clearly a prerequisite for the virulence of *F. tularensis* spp., and a locus essential for growth is the FPI. Thus, similar to the Δ*dotU* and Δ*vgrG* mutants studied here, mutations within the *iglABCD* operon of the FPI render the normally highly virulent strain Schu S4 essentially avirulent, confined to the phagosome, and unable to multiply within target cells [66], [67]. Importantly, this null phenotype is not found for all mutants within the FPI as evidenced by our demonstration that Δ*pdpE* and Δ*iglG* both exhibited intact intramacrophage multiplication, while replication of Δ*iglI* was cell-type dependent. Moreover, although Δ*iglG* and Δ*iglI* were markedly attenuated in *vivo*, Δ*pdpE* was found to be fully virulent [22], [23]. Some FPI gene products show modest homologies to conserved T6SS components, *e.g.*, IglA, IglB, IcmF, VgrG and DotU [15], [22], however, there is no evidence that this extends to functional conservation. Together, this emphasizes the importance of careful analysis to determine the roles of the FPI proteins.

The FPI proteins are highly conserved between different subspecies of *F. tularensis* and therefore the less virulent LVS strain is an appropriate model to investigate the functions of the proteins. Like all of the DotU homologues hitherto described, the *F. tularensis* DotU was found to be essential for the pathogenesis of the bacterium. As many other FPI mutants, Δ*dotU* failed to escape from the phagosome, lacked intramacrophage replication, and was essentially avirulent in the mouse model. Moreover, as confinement to the phagosome has been strongly linked to a lack of cytopathogenic effects on the host cell, the mutant caused no LDH release. Additionally, it failed to inhibit LPS-induced TNF-α secretion, and showed no activation of the inflammasome. This phenotype strongly resembles those previously described for several other FPI mutants, for example, those affecting the expression of any of the genes of the *iglABCD* operon [14], [23], [68], [69]. Probably, all of the phenotypic characteristics relate to the mutants' inability to escape from the phagosome. Moreover, the lack of intramacrophage replication likely renders the bacterium avirulent, although it has been suggested that also replication in non-macrophages could contribute to the pathogenesis of *F. tularensis* [70].

Our findings do not identify the exact role of DotU, but they do provide important clues. The normal membrane permeability and LPS profile of a Δ*dotU* mutant together with its normal adaptation to stress indicate that this inner membrane protein is dispensable for membrane integrity and plays no role in the bacterial stress response. Thus, it appears likely that the essential role of DotU in *F. tularensis* virulence is directly linked to the bacterium's intracellular life cycle. Therefore, the interaction(s) between DotU and other proteins of the putative T6SS was of definite interest to investigate. Intriguingly, we observed a post-transcriptional degradation of full-length IcmF in the Δ*dotU* mutant, similar to what has been shown for Δ*dotU* mutants of *F. novicida* and *A. tumefaciens* [12], [33]. In the *Legionella* T4SS, however, IcmF is stably expressed in the absence of DotU, whereas DotU becomes degraded in the absence of the former protein [8]. Thus, the role of DotU in T6S may be distinct from its role in T4S. Moreover, in *Legionella*, it has been suggested that DotU/IcmF forms a complex that prevents degradation of other Dot proteins of the T4SS. Besides IcmF, the levels of 7 other FPI proteins, including VgrG, were not affected by the loss of DotU in LVS, although we cannot rule out that DotU may affect the stability of other FPI proteins against which we are currently lacking antibodies. In the T6SSs of *A. tumefaciens* and *E. tarda*, IcmF and DotU proteins physically interact [11], [12]. However, we observed no such interaction for the *Francisella* homologues using either the B2H- or the Y2H system, suggesting that the interaction may be indirect. In fact, DotU did not bind to any of 12 other FPI proteins tested in these systems, *i.e.* IglA, IglB, IglC, IglD, PdpE, IglJ, IglI, IglH, IglG, IglF, VgrG and IglE (Lavander, Bröms and Meyer, unpublished results). Thus, the mechanism behind DotU-mediated stabilization of IcmF is still unclear, although their mutual localization to the inner membrane is likely to be a prerequisite.

In the *Legionella* T4SS, *dotU* and *icmF* are located adjacent on the chromosome. This linkage is also conserved in some T6S clusters, including that of *R. leguminosarum*, *P. aeruginosa* HSI-I to III, and 5 out of 6 clusters of *Burkholderia* spp. [41], [71]. In an even larger number of T6SSs, however, *dotU* is located directly adjacent to a gene, which encodes a hypothetical protein with unknown function (COG3522) [4]. This is the case for non-pathogenic bacteria like *Marinobacter* and *R. leguminosarum*, as well as bacteria pathogenic to humans and animals (*e.g. V. cholerae*, *E. coli*, *Y. pestis*, *S. typhimurium*, *Aeromonas* and *Photorhabdus*), or plants (*e.g. A. tumefaciens*, *Ralstonia solanacearum*, *Erwinia* and *Xanthomonas*) [5], [41]. Intriguingly, the DotU-COG3522 linkage is conserved in all of 3 evolutionary distinct T6SS loci of *P. aeruginosa* [41], all of 4 T6S clusters of *Salmonella* spp. [72], and all of 6 T6SS loci of *Burkholderia* spp. [71]. This strongly suggests that these two genes may co-function in T6S. In many of the aforementioned species, *dotU* is also located directly adjacent to a gene encoding ClpV (COG0542) [5], [41], [71], [72], which belongs to the AAA+ family of ATPases hypothesized to energize the T6SS [9], [16]. Interestingly, in *F. tularensis*, no homologue of either of COG3522 or COG0542 exists. Moreover, *dotU* is also localized far away from *icmF/pdpB* within the FPI. What this means for DotU function is not known. Intriguingly, based on the presence of conserved domains, five distinct groups could be identified within the DotU superfamily of proteins (DUF2077). Of these, the two largest groups consists of DotU proteins that carry an OmpA-motif, known to bind peptidoglycan, while members of the other group, *e.g. F. tularensis* DotU, lack this domain. It is tempting to speculate that this could reflect an adaptation of DotU proteins to perform different functions in T6SSs. Regardless of group belonging, the vast majority of DotU proteins were found to possess three strikingly conserved residues; in *F. tularensis* DotU these correspond to D70, E71 and G134. Substitutions of any of these residues had a major impact on DotU function with regard to its ability to support intracellular growth, cytopathogenicity, inhibition of TNF-α secretion, IcmF stability, and virulence in mice. How these residues affect DotU function is not known, but their role cannot be ascribed solely to maintaining DotU protein stability as only one out of the six substitution mutants appeared less stable.

VgrG together with Hcp are the most important secreted effectors of T6SSs. The former shows structural resemblance to the spike complex of T4 bacteriophages and some VgrG variants harbor C-terminal effector domains, such as the RtxA actin cross-linking domain of *V. cholerae* VgrG1 and the actin-ADP ribosylating domain of *A. hydrophila* VgrG1 [20], [21]. In comparison to the more well-studied homologues, *F. tularensis* VgrG is much smaller and there is no bioinformatic indication that its C-terminus harbors an active domain [22]. Thus, it appears unlikely that *F. tularensis* VgrG shows functional conservation with the characterized homologues. Nonetheless, we and others have previously demonstrated VgrG secretion during infection with LVS or *F. novicida* and this occurred also in various FPI mutant backgrounds [22], [23]. As has been postulated for the VgrGs of *V. cholerae* and *P. aeruginosa* [20], [24], [60], the *F. tularensis* protein formed multimeric complexes, consistent with its suggested role as a trimeric membrane puncturing device. Currently, we are trying to understand the nature of this interaction and if it has any biological role. Since *F. tularensis* VgrG is predicted to contain beta-strands, it is possible that extensive beta-sheet interactions may form between VgrG proteins, analogously to what has been shown for the corresponding molecular needle ( = gp5, C-terminal domain) of bacteriophage T4 [38].

Similar to DotU, the VgrG protein was essential for the intracellular life cycle and virulence of *F. tularensis*. In fact, the two mutants showed essentially identical phenotypes in the various assays investigated, including a confinement to the phagosome, a failure to multiply intracellularly, and a lack of cytopathogenicity. Additionally, the mutants did not activate the inflammasome and were markedly attenuated in the mouse model. The strong conservation of DotU and VgrG proteins in T6S clusters and their essential role in bacterial pathogenesis suggest that they are core components of T6SSs. Our ongoing work will be to define what their roles are in *F. tularensis* LVS and how this relates to the overall function of the enigmatic FPI.

## Materials and Methods

### Bacterial strains, plasmids and growth conditions

Bacterial strains and plasmids used in this study are listed in Table S1. *Escherichia coli* strains were cultured in Luria Bertani broth (LB) or on Luria agar plates at 37°C. *F. tularensis* was grown on modified GC-agar base or in liquid Chamberlain's medium [77] at 37°C. When necessary, carbenicillin (Cb; 100 µg/ml), tetracycline (Tet; 10 µg/ml), kanamycin (Km; 50 µg/ml for *E. coli*, 10 µg/ml for *F. tularensis*), or chloramphenicol (Cm; 25 µg/ml for *E. coli*, 2.5 µg/ml for *F. tularensis*) were used. For in *vitro* growth experiments of *F. tularensis*, strains were grown over night in Chamberlain's medium at 37°C, 200 rpm with good aeration. Next day, bacteria were subcultured to OD_600_ = 0.15 and grown for an additional 24 h, during which OD_600_ was measured at different time points. For pH stress experiments, *F. tularensis* was subcultured into Chamberlain's medium adjusted to pH 4 or pH 7. For H_2_O_2_ stress experiment, *F. tularensis* was subcultured into Chamberlain's medium and grown for an additional 2 h before bacteria were diluted in PBS containing different concentrations of H_2_O_2_ (0; 0,1 and 2 mM). After a 2 h incubation period, dilution series were generated and spread on modified GC-agar base plates to determine bacterial counts.

### Multidrug sensitivity assay

The multidrug sensitivity assay was adapted from Gil and colleagues [54]. Strains of *F. tularensis* were grown over night on modified GC-agar base, suspended to OD_600_ = 1 in PBS. From a 100× dilution, 100 µL of the bacterial suspension was spread on a new plate to obtain a bacterial lawn. Sterile disks (Fluka, Germany) soaked with different drugs (EtBr: 10 µg, SDS: 750 µg, or vancomycin: 100 µg) were placed on the plates. After 3 days, the growth inhibition halos around the disks were measured as the diameter of the zone of inhibition including the diameter of the disk (10 mm). The experiments were repeated at least two times for each drug and strain and in duplicate samples.

### Construction of *dotU* and *vgrG* null mutants in *F. tularensis* LVS, and complementation of *vgrG* in *cis*

Primer combinations used to construct the *dotU* and *vgrG* null mutants in LVS as well as the *cis*-complemented *vgrG* mutant are listed in Table S2. All amplified fragments were first cloned into pCR4-TOPO TA cloning vector (Invitrogen AB) to facilitate sequencing (Eurofins MWG Operon) before proceeding with the cloning. The *dotU* deletion construct was made as follows: upstream and downstream flanking regions of ∼1,200 bp were sequentially cloned into pBluescript SK+ (Stratagene, La Jolla, CA, USA) using the *Xho*I/*Bam*HI and *Bam*HI/*Sac*I sites, respectively, thereby generating a fragment encoding DotU Δ4-203 with flanking regions joined by a *Bam*HI site. The deletion fragment was cloned into *Xho*I/*Sac*I-digested pDM4 [76], generating pJEB752 (pΔ*dotU*). The *vgrG* deletion construct encoding VgrG Δ4-162 with flanking regions of ∼1,000 bp was constructed by overlap PCR [78] and lifted into *Xho*I/*Sac*I-digested pDM4, generating pMOL72 (pΔ*vgrG*). The *vgrG cis*-complementation construct was generated as follows: a ∼2,500 bp fragment incorporating *vgrG* and flanking regions was amplified by PCR, and lifted into *Xho*I/*Sac*I-digested pDM4, generating pJEB926 (pVgrG*cis*). Conjugal mating experiments using S17-1λ pir [73] as the donor strain and sucrose-selection allowed for the allelic exchange of the suicide plasmids within regions of complementary sequence on the chromosome of LVS (for null mutants) or Δ*vgrG* (for in *cis*-complementation of Δ*vgrG*) as described previously [75]. This resulted in strain Δ*vgrG*/VgrGcis, for which one of the *vgrG* deletion copies had been replaced by full-length *vgrG*. To remove both copies of the *dotU* and *vgrG* genes, the procedure was repeated, resulting in the null mutants henceforth designated Δ*dotU* and Δ*vgrG*. In all cases, PCR screening was used to verify that the anticipated genetic event had occurred.

### Construction of expression vectors

Plasmids used in this study are listed in Table S1. Primer combinations and restriction enzymes used to generate the plasmids are listed in Table S2. All amplified fragments were first cloned into pCR4-TOPO TA cloning vector to facilitate sequencing. LVS chromosomal DNA was commonly used as template in the PCR reactions. When *dotU* and *icmF* were cloned by *Nde*I digestion, the templates used were *dotU* and *icmF* alleles engineered by overlap PCR to lack their intrinsic *Nde*I-sites. For these templates, the GCA codon incorporating part of the *Nde*I site of *dotU* (nucleotides 200–205) was altered to GCT, while the ACA codon of the *Nde*I site of *icmF* (nucleotides 1091–1096) was mutated to ACT. Overlap PCR was also used to introduce substitution mutations within conserved residues of *dotU*.

Plasmids used for complementation in *trans* were constructed as follows: PCR amplified *vgrG* and *dotU* were cloned into the *Nde*I/*Eco*RI sites of pKK289Km to allow constitutive expression from the *groE* promoter [68]. The same strategy was employed for C-terminally GSK (Glycogen synthase kinase)-tagged versions of *iglA* and *iglC*, while GSK-tagged *vgrG*, *dotU* and mutated variants thereof, *iglB*, and *iglD* were constructed by introducing non-tagged alleles into the *Nde*I/*Kpn*I sites of pMOL52, a derivative of pKK289Km expressing GSK [14]. Plasmids were transferred into *F. tularensis* by electroporation. For the Bacterial-2-hybrid assay, PCR amplified *vgrG*, *dotU*, *icmF*, *iglA*, *iglB* and *iglC* were introduced as *Nde*I/*Not*I fragments into the IPTG-inducible plasmids pACTR-AP-Zif and pBRGPω [61]. Plasmids were transferred into *E. coli* DH5αF′IQ (Invitrogen AB, Stockholm, Sweden) by electroporation. For the Yeast-2-hybrid assay, PCR amplified *vgrG*, *dotU* and *icmF* were introduced into the *Nde*I/*Eco*RI sites (*vgrG*, *dotU*) or *Nde*I/*Xma*I sites (*icmF*) of the GAL4 activation domain plasmid pGADT7 or the GAL4 DNA-binding domain plasmid pGBKT7 (Clontech Laboratories, Palo Alto, CA, USA). Transformation of the *Saccharomyces cerevisiae* reporter strain AH109 was performed according to established methods [79].

### Western blot analysis

Unless stated otherwise, bacterial lysates were prepared in Laemmli sample buffer and boiled prior to separation on 10–12% sodium dodecyl sulfate (SDS)-polyacrylamide gels. Proteins were transferred onto nitrocellulose membranes using a semidry blotter (Bio-Rad laboratories, CA, USA). Membranes were probed with mouse monoclonal antibodies recognizing IglB, IglC, PdpA or IcmF/PdpB, and rabbit polyclonal antibodies recognizing IglA (all from BEI Resources, Manassas, VA, USA). To detect IglH or VgrG, rabbit polyclonal antibodies raised against the specific proteins were used (Inbiolabs, Tallinn, Estonia), while detection of IglD required chicken IgY anti-IglD (Agrisera, Vännäs, Sweden). GSK-tagged variants of DotU, VgrG, IglA, IglB, IglC, IglD were detected using rabbit anti-GSK antibodies (Cell Signaling Technology, Danvers, MA, USA). To analyze outer membrane profiles of Δ*dotU* and Δ*vgrG* mutant bacteria, outer membrane fractions prepared as described in “Fractionation of *F. tularensis*” were probed with mouse antibodies raised against *Francisella* LPS (Roland Grunow, Institute of Microbiology, Federal Armed Forces Medical Academy, Germany). The secondary horseradish peroxidase (HRP)-conjugated antibodies used were: goat anti-mouse (Santa Cruz Biotechnology, CA, USA), donkey anti-rabbit (GE Healthcare, UK) and rabbit anti-chicken IgY (Sigma-Aldrich, St. Louis, MO). For detection, the Enhanced Chemiluminescence system (ECL) (Amersham Biosciences, Uppsala, Sweden) was used.

### Outer membrane preparations of *F. tularensis*

The preparation of outer membrane fractions of *F. tularensis* has been described in detail elsewhere [23]. Protein concentrations were determined using a Nanodrop ND-1000 spectrophotometer (Thermo Fisher Scientific, DE, USA) and 5–20 µg of each fraction was separated by SDS-PAGE and analyzed using appropriate antisera and standard Western blot techniques (above).

### The Bacterial-2-hybrid assay

As the reporter strain for the bacterial-2-hybrid experiments, the *E. coli* strain KDZif1ΔZ was used. It harbors an F9 episome containing the *lac* promoter-derivative p*lac*Zif1–61 driving expression of a linked *lacZ* reporter gene [74]. Cells were grown with aeration at 37°C in LB supplemented with 0.4 mM IPTG (Isopropyl β-D-1-thiogalactopyranoside). Cells were permeabilized with SDS-CHCl_3_ and assayed for β-galactosidase (β-gal) activity as described previously [80]. Assays were performed at least three times in duplicate on separate occasions.

### The Yeast-2-hybrid assay

Protein expression analysis of yeast lysates and analysis of protein-protein interactions were performed according to established methods [79]. Yeast was grown at 30°C on synthetic dropout minimal agar (Clontech Laboratories) devoid of tryptophan and leucine (SD-LT). The interactive potential was tested by induction of two independent reporter genes: *ADE2*, by growing yeast on SD-LT agar lacking adenine (SD-LTA), and *HIS3*, by growing yeast on SD-LT agar lacking histidine (SD-LTH). Protein expression was verified using antibodies recognizing the activation or DNA-binding domain of GAL4 (Clontech Laboratories).

### Quantitative real-time PCR

Protocols for isolation of bacterial RNA, cDNA synthesis and qPCR have been described in detail elsewhere [14]. Primers used are listed in Table S2. For all samples, controls were made with either template or superscript omitted during cDNA synthesis. All reactions were performed in triplicate on three independent RNA preparations, with a 7900HT Sequence Detection System (Applied Biosystems) using the Sequence Detection System software. Samples were normalized against the *F. tularensis* 17-kDa house-keeping gene *tul4* (FTL0421) and compared to respective genes in LVS. Results were analyzed using the delta delta Ct method of analysis and converted to relative expression ratio (2^−ΔΔCt^) for statistical analysis [81]. Paired two-tailed t-tests were used to compare means.

### Cultivation and infection of macrophages

J774 macrophages were used in all cell infection assays, except for the IL-1β secretion assay where mouse peritoneal macrophages (PECs) were used. J774 macrophages were cultured and maintained in DMEM (GIBCO BRL, Grand Island, NY, USA) with 10% heat-inactivated FBS (GIBCO). PECs were isolated from 8- to 10-week-old C57BL/6 mice 3 days after intraperitoneal injection of 2 ml of 10% proteose peptone as previously described [82]. The day before infection, macrophages were seeded in tissue culture plates in DMEM with 10% FBS. Following incubation overnight, cells were washed, reconstituted with fresh culture medium and allowed to recover for at least 30 min prior to infection. A multiplicity of infection (MOI) of 200 was used in all infection experiments, with the exception of the TNF-α secretion assay where we used a MOI of 500 [49] and for the TEM study, where a MOI of 1000 was used [23].

### Intracellular replication in macrophages

To determine the ability of *F. tularensis* to grow within macrophages, cells were infected for 2 h, washed three times, and incubated in the presence of 5 µg/ml gentamicin for 30 min (corresponds to time zero). At 0, 24 and 48 h, the macrophage monolayers were lysed in PBS with 0.1% deoxycholate, serially diluted in PBS and plated on modified GC-agar base plates for determination of viable counts. A two-sided *t*-test with equal variance was used to determine whether the growth of a strain differed significantly from that of LVS.

### LDH release assay

The LDH release assay has been described in detail elsewhere [23]. In short, cells were infected as described in “Intracellular replication in macrophages” and supernatants were sampled at 0, 24 or 48 h and assayed for the presence of released Lactate dehydrogenase (LDH). Data are means ± standard deviations of three wells from one representative experiment of three. Uninfected cells lysed in PBS with 0.1% deoxycholate served as a positive control, and the value for this control was arbitrarily considered 100% cell lysis. Sample absorbance was expressed as the percentage of the positive control value.

### Intracellular immunofluorescence assay

To assess phagosomal escape, GFP-expressing *F. tularensis* were used in the cell infections as described previously [14]. Cells were then stained for the LAMP-1 glycoprotein as described previously [68]. Colocalization of GFP-labeled *F. tularensis* and LAMP-1 was analyzed with an epifluorescence microscope (ZeissAxioskop2; Carl Zeiss MicroImaging GmbH, Germany) and a confocal microscope (Nikon Eclipse 90i, Nikon, Japan). From two separate experiments, each with a total number of 5 glass slides per strain, 50 bacteria/slide were scored. To verify that the colocalization level was significantly different from that of LVS, a Wilcoxon Rank-sum test was used.

### Transmission electron microscopy

The protocol describing the infection and sample preparation for TEM has been described in details elsewhere [23]. Sections were viewed with a JEOL JEM 1230 Transmission Electron Microscope (JEOL Ltd., Tokyo, Japan). To examine membrane integrity, at least 100 bacteria from two different sections were analyzed and categorized as having: (i) an intact phagosomal membrane, (ii) a slightly damaged phagosomal membrane (<50% of membrane integrity affected), (iii) a highly damaged phagosomal membrane (>50% of membrane integrity affected) or (iv) no residual membrane.

### TNF-α secretion assay

To measure TNF-α secretion upon 2 h of LPS stimulation of J774 cells, we followed our previously established protocols [23].

### IL-1β secretion assay

The IL-1β secretion assay was performed as described previously [23]. Samples were taken at 5 or 24 h and analyzed using the BD OptEIA Mouse IL-1β Elisa Set (BD Biosciences) according to the manufacturer's instructions.

### Mouse infections

For determination of the killing capacity of each strain, C57BL/6 female mice (n = 5) were infected intradermally. Aliquots of the diluted cultures were also plated on GC-agar to determine the number of CFU injected. For one experiment in which approximately 4×10^7^ or 6×10^8^ CFU of *F. tularensis* were injected, the actual doses were: 4.2×10^7^ (LVS), 6.6×10^8^ (Δ*vgrG*), 4.6×10^8^ (Δ*vgrG*/pMOL54), 6.2×10^8^ (Δ*dotU*) and 4.1×10^7^ or 7.5×10^8^ (Δ*dotU*/pMOL58). In a second experiment, where approximately 5×10^7^ or 10×10^8^ CFU of *F. tularensis* was injected, the doses were: 5.3×10^7^ (LVS), 15.3×10^8^ (Δ*dotU*), 11.9×10^8^ (Δ*dotU*/pMOL58), 10.2×10^8^ (Δ*dotU*/pJEB916), 7.3×10^8^ (Δ*dotU*/pJEB917) and 11.4×10^8^ (Δ*dotU*/pJEB918). Mice were examined twice daily for signs of severe infection and euthanized by CO_2_ asphyxiation as soon as they displayed signs of irreversible morbidity. In our experience, such mice were at most 24 h from death, and time to death of these animals was estimated on this premise. To estimate the bacterial burden in organs, spleens were isolated at day 12 p.i., homogenized in PBS and aliquots plated on GC-agar to determine the number of viable bacteria. All animal experiments were approved by the Local Ethical Committee on Laboratory Animals, Umeå, Sweden (no. A113-08).

### Bioinformatic and phylogenetic analysis of Type VI Secretion Systems

The conserved domain architecture retrieval tool (CDART) [39] was used to identify homologues of *F. tularensis* DotU and to investigate DUF2077 superfamily domain architectures. Based on a length criterion aimed at selecting for full length proteins, a total of 653 DotU homologues were selected for further analysis. For single-domain proteins (contain DUF2077 domain only), only proteins longer than 200 amino acids were included, and for two-domain proteins (contain DUF2077 domain and an additional OmpA or SPOR domain) only proteins longer than 350 amino acids were included. The selected homologues were aligned using MSAprobs v. 0.9.5 [47] using 50 iterative refinement repetitions and two consistency repetitions. The conservation of the Asp70, Glu71 and Gly134 residues of the *F. tularensis* DotU within the dataset was investigated by visual inspection of the alignment. For efficient inference of phylogenetic relationships between the DotU homologues, the number of aligned sequences was reduced further using T-Coffee v. 8.99 [83], by including all *Francisella* DotU homologues but excluding all non-*Francisella* DotU homologues that exhibited more than 80% amino acid identity to any other homologue in the dataset. Thereby, a final dataset containing 283 amino acid sequences was obtained and used to determine the phylogenetic relationship among DotU proteins, which was conducted using MEGA 5.05 [46]. Phylogenetic analysis was performed using the neighbor-joining algorithm [43] and the Jones-Taylor-Thornton substitution model (JTT) with the pairwise deletion option [45]. Bootstrap analysis was performed using 100 repetitions [44]. The existence of remote homologues to *F. tularensis* DotU and VgrG proteins was investigated using the HHpred [37] and Phyre2 [36] tools, which are based on comparison of profile hidden Markov models and sequence profiles, respectively, making use of secondary structure information.

## Supporting Information

Figure S1 **Evolutionary relationships of DotU homologues.** The evolutionary history was inferred using the Neighbor-Joining method [43]. The percentage of replicate trees in which the associated taxa clustered together in the bootstrap test (100 replicates) is shown next to the branches [44]. The tree is drawn to scale, with branch lengths in the same units as those of the evolutionary distances used to infer the phylogenetic tree. The evolutionary distances were computed using the JTT matrix-based method [45] and are in the units of the number of amino acid substitutions per site. The analysis involved 283 amino acid sequences. There were a total of 870 positions in the final dataset. Evolutionary analyses were conducted in MEGA5 [46].
(PDF)

Figure S2 **Sequence comparison of DotU homologues from different bacterial species.** Alignments were generated using the ClustalW2 web server (http://www.ebi.ac.uk/Tools/clustalw2/index.html) and areas of amino acid identity (black boxes) or similarity (grey boxes) illustrated using the BOXSHADE 3.21 web server (http://www.ch.embnet.org/software/BOX_form.html). Bacterial strains (and protein IDs) are as follows: *Vibrio cholerae* O1 biovar El Tor str. N16961 (VCA0115); *Marinomonas* sp. MWYL1 (Mmwyl1_1204); *Shewanella woodyi* ATCC 51908 (Swoo_2521); *Escherichia coli* O157:H7 str. Sakai (ECs0224); *Shigella sonnei* Ss046 (SSON_0244); *Yersinia pestis* CO92 (YPO3598); *Photorhabdus asymbiotica* subsp. *asymbiotica* ATCC 43949 (PAU_00280); *Proteus mirabilis* HI4320 (PMI0741); *Aeromonas hydrophila* subsp. *hydrophila* ATCC 7966 (AHA_1840); *Pectobacterium wasabiae* WPP163 (Pecwa_1078); *Pseudomonas aeruginosa* PA01 (PA1668); *Ralstonia solanacearum* GMI1000 (RS01969); *Cupriavidus taiwanensis* LMG 19424 (RALTA_B1009); *Burkholderia pseudomallei* K96243 (BPSL3111); *Dechloromonas aromatica* RCB (Daro_2181); *Xanthomonas oryzae* pv. oryzae KACC10331 (XOO3485); *Acinetobacter* sp. ADP1 (ACIAD2697);. *Azoarcus* sp. BH72 (azo1298); *Chromobacterium violaceum* ATCC 12472 (CV_3984); *Pseudomonas putida* F1 (Pput_2630); *Candidatus Solibacter* usitatus Ellin6076 (Acid_0224); *Edwarsiella tarda* PPD130/91 (EvpN); *Pseudomonas aeruginosa* PA01 (PA2362), *Legionella pneumophila* Philadelphia-1 (DotU); *Desulfonatronospira thiodismuta*ns ASO3-1 (DotU); *Francisella tularensis* subsp. *holarctica* LVS (FTL_0119/DotU).
(DOCX)

Table S1 **Strains and plasmids used in this study.**
(DOCX)

Table S2 **Oligonucleotides used in this study.**
(DOCX)
