# Supplementary figures and images for: DotU and VgrG, Core Components of Type VI Secretion Systems, Are Essential for Francisella LVS Pathogenicity
Source: PLoS One. 2012 Apr 13;7(4):e34639. doi: 10.1371/journal.pone.0034639 (PMC3326028; doi:10.1371/journal.pone.0034639)

Figure S1

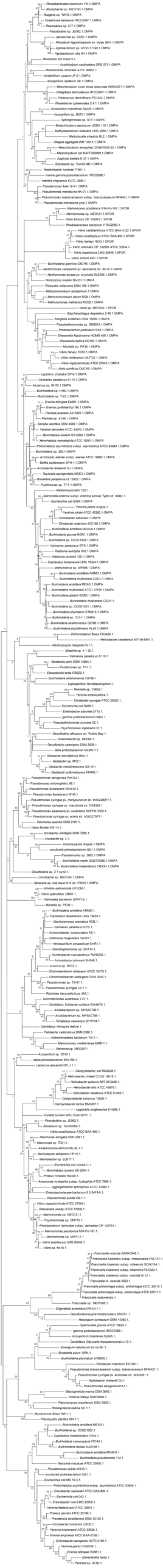

Supplement: Figure S1 — Evolutionary relationships of DotU homologues. The evolutionary history was inferred using the Neighbor-Joining method [43]. The percentage of replicate trees in which the associated taxa clustered together in the bootstrap test (100 replicates) is shown next to the branches [44]. The tree is drawn to scale, with branch lengths in the same units as those of the evolutionary distances used to infer the phylogenetic tree. The evolutionary distances were computed using the JTT matrix-based method [45] and are in the units of the number of amino acid substitutions per site. The analysis involved 283 amino acid sequences. There were a total of 870 positions in the final dataset. Evolutionary analyses were conducted in MEGA5 [46]. (PDF) [file pone.0034639.s001.pdf]
