# Supplementary material for: DotU and VgrG, Core Components of Type VI Secretion Systems, Are Essential for Francisella LVS Pathogenicity
Source: PLoS One. 2012 Apr 13;7(4):e34639. doi: 10.1371/journal.pone.0034639 (PMC3326028; doi:10.1371/journal.pone.0034639)
Supplement: Figure S2 — Sequence comparison of DotU homologues from different bacterial species. Alignments were generated using the ClustalW2 web server (http://www.ebi.ac.uk/Tools/clustalw2/index.html) and areas of amino acid identity (black boxes) or similarity (grey boxes) illustrated using the BOXSHADE 3.21 web server (http://www.ch.embnet.org/software/BOX_form.html). Bacterial strains (and protein IDs) are as follows: Vibrio cholerae O1 biovar El Tor str. N16961 (VCA0115); Marinomonas sp. MWYL1 (Mmwyl1_1204); Shewanella woodyi ATCC 51908 (Swoo_2521); Escherichia coli O157:H7 str. Sakai (ECs0224); Shigella sonnei Ss046 (SSON_0244); Yersinia pestis CO92 (YPO3598); Photorhabdus asymbiotica subsp. asymbiotica ATCC 43949 (PAU_00280); Proteus mirabilis HI4320 (PMI0741); Aeromonas hydrophila subsp. hydrophila ATCC 7966 (AHA_1840); Pectobacterium wasabiae WPP163 (Pecwa_1078); Pseudomonas aeruginosa PA01 (PA1668); Ralstonia solanacearum GMI1000 (RS01969); Cupriavidus taiwanensis LMG 19424 (RALTA_B1009); Burkholderia pseudomallei K96243 (BPSL3111); Dechloromonas aromatica RCB (Daro_2181); Xanthomonas oryzae pv. oryzae KACC10331 (XOO3485); Acinetobacter sp. ADP1 (ACIAD2697);. Azoarcus sp. BH72 (azo1298); Chromobacterium violaceum ATCC 12472 (CV_3984); Pseudomonas putida F1 (Pput_2630); Candidatus Solibacter usitatus Ellin6076 (Acid_0224); Edwarsiella tarda PPD130/91 (EvpN); Pseudomonas aeruginosa PA01 (PA2362), Legionella pneumophila Philadelphia-1 (DotU); Desulfonatronospira thiodismutans ASO3-1 (DotU); Francisella tularensis subsp. holarctica LVS (FTL_0119/DotU). (DOCX) [file pone.0034639.s002.docx]

**Figure S2**

| V_cholerae | - | - | - | - | - | - | - | - | - | - | - | - | - | - | - | - | - | - | - | - | - | - | - | - | M | S | Q | S | K | K | E | T | P | L | A | S | L | L | F | D | D | V | E | K | I | N | H | D | Q | D | Y | W | F | Q | L | R | G | D | N | P |
| --- | --- | --- | --- | --- | --- | --- | --- | --- | --- | --- | --- | --- | --- | --- | --- | --- | --- | --- | --- | --- | --- | --- | --- | --- | --- | --- | --- | --- | --- | --- | --- | --- | --- | --- | --- | --- | --- | --- | --- | --- | --- | --- | --- | --- | --- | --- | --- | --- | --- | --- | --- | --- | --- | --- | --- | --- | --- | --- | --- | --- |
| Marinomonas | - | - | - | - | - | - | - | - | - | - | - | - | - | - | - | - | - | - | - | - | - | - | - | - | M | A | S | N | G | I | I | G | K | Y | D | D | L | L | F | D | E | A | E | N | I | N | D | D | K | D | Y | W | F | Q | I | K | G | H | N | D |
| S_woodyl | - | - | - | - | - | - | - | - | - | - | - | - | - | - | - | - | - | - | - | - | - | M | K | P | Q | K | Q | P | L | T | E | Q | Q | L | D | D | V | L | F | D | E | V | R | N | I | D | A | D | K | E | Y | W | F | Q | L | R | G | D | N | I |
| E_coli | - | - | - | - | - | - | - | - | - | - | - | - | - | - | - | - | - | - | - | - | - | - | - | - | - | - | - | - | - | M | D | E | G | S | L | S | L | P | P | F | T | G | Y | D | E | K | S | L | R | D | Y | H | L | A | L | H | G | N | S | L |
| S_sonnei | - | - | - | - | - | - | - | - | - | - | - | - | - | - | - | - | - | - | - | - | - | - | - | - | - | - | - | - | - | M | D | E | G | S | L | S | L | P | P | F | T | G | H | D | E | K | S | Q | R | N | Y | H | L | A | L | R | G | N | S | L |
| Y_pestis | - | - | - | - | - | - | - | - | - | - | - | - | - | - | - | - | - | - | - | - | - | - | - | - | - | - | M | K | T | D | A | V | T | D | N | T | P | F | P | A | A | E | A | V | T | S | - | H | R | Q | Y | Q | L | P | L | R | G | E | S | L |
| P_asymbiotica | - | - | - | - | - | - | - | - | - | - | - | - | - | - | - | - | - | - | - | - | - | - | - | - | - | - | M | K | N | E | A | I | N | D | N | A | I | L | M | Q | V | E | T | D | T | T | L | H | R | Q | Y | Q | L | P | L | R | G | E | S | L |
| P_mirabilis | - | - | - | - | - | - | - | - | - | - | - | - | - | - | - | - | - | - | - | - | - | - | - | - | - | - | - | - | - | - | - | M | T | D | D | I | L | L | N | H | D | D | E | E | R | I | T | K | K | N | Y | Q | L | P | L | R | G | N | N | I |
| A_hydrophila | - | - | - | - | - | - | - | - | - | - | - | - | - | - | - | - | - | - | - | - | - | - | - | - | - | - | - | - | - | - | - | - | - | - | - | - | - | - | - | - | - | - | - | - | - | - | M | D | S | D | Y | W | F | R | L | R | G | Q | S | I |
| P_wasabiae | - | - | - | - | - | - | - | - | - | - | - | - | - | - | - | - | - | - | - | - | - | - | - | M | S | I | E | V | I | K | N | D | Q | L | G | D | L | L | F | D | H | A | R | Q | L | D | M | D | S | D | Y | W | F | R | L | R | G | Q | S | I |
| P_aeruginosa_HSI-II | M | I | K | E | R | E | Y | H | E | D | E | K | T | V | L | L | D | H | Q | G | Q | V | P | A | Q | G | P | L | T | D | F | A | E | P | P | R | F | E | Q | L | E | E | R | M | I | Y | A | A | R | L | R | P | G | E | A | F | N | V | S | L |
| R_solanacearum | - | - | - | - | - | - | - | - | - | - | - | - | - | - | - | - | - | - | - | - | - | - | - | - | - | - | - | - | M | S | A | T | T | T | P | S | L | F | G | G | S | - | A | A | P | A | G | N | A | A | A | D | A | R | E | S | N | T | H | V |
| C_taiwanensis | - | - | - | - | - | - | - | - | - | - | - | - | - | - | - | - | - | - | - | - | - | - | - | - | - | - | - | - | M | S | A | T | S | T | P | S | L | F | G | A | S | P | A | G | Q | P | S | V | A | A | A | G | S | A | D | G | A | M | H | A |
| B_pseudomallei | - | - | - | - | - | - | - | - | - | - | - | - | - | - | - | - | - | - | - | - | - | - | - | - | - | - | - | - | - | - | M | S | Y | A | P | S | L | F | G | G | N | - | T | P | P | P | A | P | Q | T | A | S | S | T | D | A | G | F | Q | A |
| D_aromatica | - | - | - | - | - | - | - | - | - | - | - | - | - | - | - | - | - | - | - | - | - | - | - | - | - | - | - | - | - | - | M | T | T | A | P | S | L | F | S | I | A | P | S | T | R | P | D | D | S | V | T | D | R | P | - | - | - | - | - | A |
| X_oryzae | - | - | - | - | - | - | - | - | - | - | - | - | - | - | - | - | - | - | - | - | - | - | - | - | - | - | - | - | - | M | M | N | P | L | P | P | T | P | G | P | M | P | S | L | T | A | Q | A | P | H | G | L | P | S | A | H | T | S | T | P |
| Acinetobacter | - | - | - | - | - | - | - | - | - | - | - | - | - | - | - | - | - | - | - | - | - | - | - | - | - | - | - | M | S | Q | P | T | G | I | P | S | L | F | D | D | G | - | - | K | I | G | G | D | F | S | P | P | N | T | Q | N | V | S | Q | A |
| Azoarcus | - | - | - | - | - | - | - | - | - | - | - | - | - | - | - | - | - | - | - | - | - | - | - | - | - | - | - | - | - | - | - | - | - | - | - | - | - | - | - | - | M | A | I | A | S | T | - | T | S | A | A | A | V | P | D | R | S | P | G | A |
| C_violaceum | - | - | - | - | - | - | - | - | - | - | - | - | - | - | - | - | - | - | - | - | - | - | - | - | - | - | - | - | - | - | - | - | - | - | - | - | - | - | - | - | M | N | Q | A | S | T | Q | T | P | E | R | A | I | T | V | S | A | P | A | A |
| P_putida | - | - | - | - | - | - | - | - | - | - | - | - | - | - | - | - | - | - | - | - | - | - | - | - | - | - | - | - | - | - | - | - | - | - | - | - | - | - | - | - | - | - | - | M | T | E | A | V | L | Q | Q | G | A | V | P | A | A | S | D | K |
| C_Solibacter | - | - | - | - | - | - | - | - | - | - | - | - | - | - | - | - | - | - | - | - | - | - | - | - | - | - | - | - | - | - | - | - | - | - | - | - | - | - | - | - | - | - | - | - | - | - | - | - | - | - | M | P | P | S | A | D | T | R | R | P |
| E_tarda | - | - | - | - | - | - | - | - | - | - | - | - | - | - | - | - | - | - | - | - | - | - | - | - | - | - | - | - | - | - | - | - | - | - | - | - | - | - | - | - | - | - | - | - | - | - | - | - | - | - | - | - | - | - | - | - | - | - | - | M |
| P_aeruginosa_HSI-III | - | - | - | - | - | - | - | - | - | - | - | - | - | - | - | - | - | - | - | - | - | - | - | - | - | - | - | - | - | - | - | - | - | - | - | - | - | M | P | E | G | S | A | G | P | F | S | Q | A | Y | Q | E | Q | P | L | S | T | A | F | R |
| L_pneumophila | - | - | - | - | - | - | - | - | - | - | - | - | - | - | - | - | - | - | - | - | - | - | M | T | T | E | Q | Y | P | S | S | L | V | N | R | L | A | I | T | E | P | A | L | V | P | S | G | Y | Y | R | S | K | L | F | I | A | P | F | S | T |
| D_thiodismutans | - | - | - | - | - | - | - | - | - | - | - | - | - | - | - | - | - | - | - | - | - | - | - | - | M | K | N | N | H | W | K | D | I | H | T | L | L | V | Q | R | D | Q | L | F | M | P | F | L | S | S | G | E | E | N | R | E | G | F | E | F |
| F_tularensis | - | - | - | - | - | - | - | - | - | - | - | - | - | - | - | - | - | - | - | - | - | - | - | - | - | - | - | - | - | - | - | - | - | - | - | - | - | - | - | - | - | - | - | - | - | - | - | - | - | - | - | - | - | - | - | - | - | M | K | D |
|  |  |  |  |  |  |  |  |  |  |  |  |  |  |  |  |  |  |  |  |  |  |  |  |  |  |  |  |  |  |  |  |  |  |  |  |  |  |  |  |  |  |  |  |  |  |  |  |  |  |  |  |  |  |  |  |  |  |  |  |  |
| V_cholerae | N | V | L | I | D | A | A | T | P | L | F | G | L | S | L | R | V | R | T | L | T | E | - | C | D | N | I | E | Q | I | Y | R | Q | T | I | E | E | I | K | A | I | E | I | E | L | T | E | Q | G | Y | E | H | A | I | L | M | A | Y | R | Y |
| Marinomonas | N | P | L | I | D | S | A | T | T | F | F | G | L | S | L | R | V | K | S | L | S | E | - | C | E | N | I | E | E | I | Y | R | Q | T | I | E | E | I | N | I | I | E | I | E | L | A | D | K | H | Y | E | H | S | V | L | M | A | Y | R | Y |
| S_woodyl | N | Q | L | I | D | A | A | T | P | L | M | G | M | V | F | R | V | R | K | L | A | V | - | L | D | D | V | Q | K | L | Y | H | N | T | V | D | D | I | M | A | I | E | A | E | L | T | E | S | G | F | G | R | A | I | I | L | A | Y | R | Y |
| E_coli | N | P | M | I | D | A | A | T | P | L | L | G | M | V | M | R | L | S | T | M | N | S | - | Q | T | M | P | E | H | L | F | A | Q | V | V | T | D | V | Q | A | V | E | Q | L | L | Q | E | Q | G | Y | E | P | G | V | I | I | S | F | R | Y |
| S_sonnei | N | P | M | I | D | A | A | T | P | L | L | G | M | V | M | R | L | S | T | M | N | S | - | Q | T | M | P | E | H | L | F | A | Q | V | V | T | D | V | Q | A | V | E | Q | L | L | Q | E | Q | G | Y | E | P | G | V | I | I | S | F | R | Y |
| Y_pestis | N | A | M | I | D | T | A | T | P | L | L | G | M | V | L | R | L | K | D | M | E | N | - | Q | A | L | P | D | Q | L | Y | Q | Q | V | V | T | D | I | R | A | I | E | Q | Y | L | Q | T | K | G | Y | E | P | G | A | I | I | S | F | R | Y |
| P_asymbiotica | N | P | M | I | D | A | A | T | P | L | L | G | M | V | M | R | L | K | D | M | G | D | - | E | A | L | P | D | Q | L | Y | Q | Q | V | V | T | D | I | Q | A | I | E | Q | F | L | Q | T | K | G | Y | E | P | G | A | I | V | S | F | R | Y |
| P_mirabilis | N | P | M | I | D | A | A | T | P | L | L | G | M | V | L | R | M | K | A | M | S | E | - | T | P | L | S | E | K | L | Y | Q | Q | V | V | M | D | I | T | S | I | E | Q | Q | L | Q | I | Q | G | Y | E | P | G | A | I | V | S | F | R | Y |
| A_hydrophila | N | P | M | I | D | A | V | T | P | L | L | G | L | V | Q | R | V | R | L | L | S | R | - | Y | D | R | V | P | E | L | Y | Q | R | V | V | T | E | I | Q | A | I | E | Q | E | L | M | A | Q | G | Y | E | N | G | V | V | L | S | F | R | Y |
| P_wasabiae | N | P | M | I | D | A | V | T | P | L | L | G | M | V | E | R | V | R | Q | L | S | A | - | Y | E | G | V | E | D | L | Y | Q | R | V | V | S | E | V | Q | A | I | E | Q | E | L | H | S | H | S | Y | E | N | G | V | I | L | S | F | R | Y |
| P_aeruginosa_HSI-II | N | P | L | V | A | A | A | S | E | L | L | S | E | V | V | R | L | K | H | S | T | V | - | L | E | N | L | A | A | L | K | E | R | L | T | S | A | M | K | L | F | E | F | R | A | V | Q | D | G | V | E | N | S | Q | V | M | A | A | R | Y |
| R_solanacearum | R | T | L | L | D | L | L | Y | D | G | F | F | M | L | F | Q | L | R | N | G | Q | Q | - | P | T | S | A | E | D | F | L | T | R | V | R | A | F | L | E | D | F | D | R | G | A | K | R | L | N | V | S | A | E | D | V | F | D | A | K | Y |
| C_taiwanensis | R | T | L | L | D | L | L | Y | D | G | F | F | M | L | F | L | L | R | N | G | Q | Q | - | P | G | S | A | E | E | F | L | Q | K | V | R | E | F | L | D | D | F | E | R | G | A | K | R | L | N | V | A | A | E | D | I | F | D | A | K | Y |
| B_pseudomallei | R | S | L | V | D | L | L | Y | D | G | F | F | M | L | F | L | L | K | N | G | R | E | - | P | N | D | A | S | E | F | G | T | R | I | Q | E | F | L | S | E | F | E | R | G | A | K | K | L | N | I | A | A | D | D | V | Y | A | A | K | F |
| D_aromatica | K | N | L | V | D | L | L | Y | D | G | F | Y | M | L | I | L | L | N | N | R | S | V | - | P | K | D | P | D | E | F | S | G | N | I | Q | K | F | L | D | Q | F | E | R | A | A | K | K | N | N | F | N | A | E | D | I | F | D | A | K | Y |
| X_oryzae | Q | S | L | L | D | L | M | A | D | G | F | Y | L | L | L | L | L | K | R | T | Q | M | - | P | S | D | T | E | S | F | V | Q | S | V | Q | T | F | L | D | G | V | E | R | G | A | V | K | L | G | I | A | S | E | D | I | Y | A | A | K | Y |
| Acinetobacter | V | N | L | V | D | L | L | H | D | G | F | Y | I | V | F | L | I | R | N | Q | Y | V | - | P | A | N | I | E | E | F | R | Q | K | I | L | D | L | L | N | R | F | E | Q | Q | A | K | K | M | Q | F | S | G | D | D | I | Y | D | A | K | Y |
| Azoarcus | T | P | L | I | E | L | L | D | D | G | F | H | L | L | A | L | L | R | Q | R | A | R | - | P | R | H | Y | D | S | F | V | D | R | V | L | A | L | L | R | D | F | E | R | N | A | L | A | A | G | K | A | P | P | E | I | E | Q | A | R | Y |
| C_violaceum | P | A | M | R | E | M | L | E | D | G | I | Y | L | L | F | L | L | K | E | G | N | A | - | P | S | S | A | V | E | F | N | R | R | V | D | H | F | L | G | Q | F | E | R | N | A | R | N | F | N | K | D | N | N | A | I | S | H | A | K | Y |
| P_putida | P | T | F | K | D | L | V | Q | D | F | I | S | M | A | L | I | V | R | R | G | R | Q | - | V | T | S | V | Q | A | F | E | G | S | V | E | R | F | F | A | N | L | E | R | D | A | R | A | A | N | Y | S | V | E | Q | V | K | D | T | Q | Y |
| C_Solibacter | E | N | L | A | L | L | F | Q | D | V | L | T | A | I | V | R | L | R | S | N | R | Q | G | V | I | D | P | A | A | F | R | H | Q | I | R | E | A | L | K | S | A | A | S | R | A | L | S | A | G | Y | T | A | D | D | A | R | H | A | T | F |
| E_tarda | S | L | S | E | A | F | I | Q | P | M | L | Y | V | R | Q | Y | L | Q | A | P | E | G | - | - | - | E | P | S | L | F | R | E | R | L | Q | S | Y | L | H | H | S | Q | L | R | A | Q | E | A | G | E | S | P | A | A | I | D | S | A | L | Y |
| P_aeruginosa_HSI-III | Q | A | W | Q | E | W | L | E | A | W | G | A | L | D | R | D | A | Q | D | V | P | R | M | V | E | R | A | L | E | L | S | T | R | I | T | R | R | L | W | R | S | A | F | - | A | S | V | G | D | A | A | G | V | Q | V | K | A | M | V | Y |
| L_pneumophila | N | V | L | V | A | A | A | G | P | I | L | S | L | L | E | R | L | C | L | S | P | S | - | L | P | P | V | E | D | I | R | E | N | I | Q | H | E | L | R | A | F | H | S | K | L | D | A | S | K | Y | P | L | D | I | I | S | I | A | Q | Y |
| D_thiodismutans | S | D | L | H | Q | D | S | Q | H | N | G | W | T | L | P | G | S | S | S | Q | E | P | G | I | E | D | L | V | Q | V | R | A | S | I | R | D | E | L | D | R | L | R | I | - | A | L | E | T | D | L | N | E | R | D | V | Y | Y | I | L | F |
| F_tularensis | F | K | E | I | E | I | I | L | D | I | I | K | T | T | R | E | I | I | E | D | N | D | N | D | N | E | K | I | S | Y | H | R | N | N | I | R | K | S | I | F | F | L | Q | E | E | L | L | E | K | Y | S | E | T | V | C | K | Y | I | V | F |
|  |  |  |  |  |  |  |  |  |  |  |  |  |  |  |  |  |  |  |  |  |  |  |  |  |  |  |  |  |  |  |  |  |  |  |  |  |  |  |  |  |  |  |  |  |  |  |  |  |  |  |  |  |  |  |  |  |  |  |  |  |
| V_cholerae | I | L | C | A | F | L | D | E | S | V | M | G | T | E | - | - | - | - | W | G | A | S | S | L | W | A | E | H | S | M | L | S | R | F | H | N | E | T | W | G | G | E | K | V | F | T | I | L | S | R | L | - | - | E | G | E | P | H | R | Y |
| Marinomonas | I | L | C | A | F | L | D | E | A | V | M | G | T | R | - | - | - | - | W | G | G | S | S | A | W | A | E | Y | S | M | L | S | R | F | H | N | E | T | W | G | G | E | K | V | F | S | I | L | S | R | L | - | - | E | K | D | P | E | K | Y |
| S_woodyl | V | L | C | S | F | I | D | E | A | V | M | N | T | P | - | - | - | - | W | G | A | D | S | V | W | A | E | H | S | L | L | T | R | F | H | N | E | T | W | G | G | E | K | V | F | S | I | L | Q | R | L | - | - | E | T | E | P | A | S | Y |
| E_coli | I | L | C | T | F | I | D | E | A | A | L | G | N | G | - | - | - | - | W | S | N | K | N | E | W | I | K | Q | S | L | L | V | H | F | H | N | E | A | W | G | G | E | K | V | F | I | L | L | E | R | L | - | - | I | R | E | P | K | R | Y |
| S_sonnei | I | L | C | T | F | I | D | E | A | A | L | G | N | G | - | - | - | - | W | S | N | K | N | E | W | I | K | Q | S | L | L | V | H | F | H | N | E | A | W | G | G | E | K | V | F | I | L | L | E | R | L | - | - | I | R | E | P | K | R | Y |
| Y_pestis | V | L | C | T | F | I | D | E | T | A | L | G | H | G | - | - | - | - | W | N | T | Q | N | G | W | L | Q | Q | S | L | L | V | H | F | H | N | E | T | W | G | G | E | K | V | Y | V | L | L | E | R | L | - | - | M | G | E | P | K | R | Y |
| P_asymbiotica | M | L | C | T | F | I | D | E | T | A | L | G | H | G | - | - | - | - | W | N | S | Q | N | G | W | L | Q | Q | S | L | L | V | Q | F | H | N | E | T | W | G | G | E | K | V | F | V | L | L | E | R | L | - | - | M | G | E | P | Q | R | Y |
| P_mirabilis | V | L | C | T | F | I | D | E | T | A | L | G | L | G | - | - | - | - | W | D | Q | D | N | G | W | V | K | Q | S | L | L | V | H | F | H | N | E | S | W | G | G | E | K | V | F | V | L | I | E | R | L | - | - | L | G | E | P | K | R | Y |
| A_hydrophila | I | L | C | T | F | I | D | E | A | V | M | G | R | D | - | - | - | - | W | G | S | Q | S | E | W | S | E | H | S | L | L | T | R | F | H | N | E | T | W | G | G | E | K | V | F | V | L | L | A | R | L | - | - | Q | E | D | P | V | R | Y |
| P_wasabiae | I | L | C | T | F | I | D | E | A | V | M | G | R | E | - | - | - | - | W | G | G | Q | S | M | W | S | A | H | S | L | L | T | R | F | H | N | E | T | W | G | G | E | K | V | F | V | L | L | E | K | L | - | - | L | D | D | P | T | R | Y |
| P_aeruginosa_HSI-II | V | L | C | T | V | V | D | E | A | V | V | T | T | P | - | - | - | - | W | G | N | E | S | E | W | S | K | I | S | L | L | S | S | F | H | N | E | T | F | G | G | E | K | F | F | Q | L | L | D | R | L | - | - | S | K | N | P | V | K | H |
| R_solanacearum | A | F | C | A | A | V | D | E | T | I | L | S | S | N | - | - | - | - | F | S | I | R | T | A | W | E | R | R | P | L | Q | L | E | L | F | G | E | Q | L | A | G | E | T | F | F | I | K | L | E | E | L | - | - | R | A | H | G | A | P | R |
| C_taiwanensis | A | F | C | A | A | I | D | E | T | I | L | A | S | N | - | - | - | - | F | S | I | R | S | T | W | E | R | R | P | L | Q | L | V | L | F | G | E | Q | L | A | G | E | G | F | F | S | K | L | E | E | L | - | - | R | A | H | G | A | P | R |
| B_pseudomallei | A | F | C | A | A | I | D | E | S | V | L | S | S | P | - | - | - | - | F | K | I | R | A | D | W | E | R | R | P | L | Q | L | V | L | F | G | E | Q | L | A | G | E | K | F | Y | Q | Y | L | E | E | C | - | - | R | A | Q | G | A | A | R |
| D_aromatica | A | F | C | A | A | I | D | E | S | V | L | S | S | R | - | - | - | - | M | N | I | R | D | V | W | E | R | R | P | L | Q | L | V | L | F | G | D | Q | L | A | G | E | H | F | F | D | K | L | E | I | A | - | - | R | N | G | G | A | S | R |
| X_oryzae | A | F | C | A | A | V | D | E | A | I | L | S | Q | P | - | - | - | - | S | A | L | H | E | T | W | E | R | N | P | L | Q | L | R | L | F | G | E | H | L | A | G | E | H | F | F | D | R | L | E | E | L | - | - | R | R | Q | G | A | V | R |
| Acinetobacter | A | F | C | A | L | L | D | E | T | I | V | T | Q | Q | D | P | N | F | F | H | L | Q | N | A | W | L | I | S | P | L | Q | L | S | L | F | G | S | Q | L | A | G | Y | R | F | F | E | I | L | E | Q | L | - | - | R | S | K | G | K | E | R |
| Azoarcus | A | F | C | A | A | L | D | E | V | V | L | S | S | D | - | - | - | - | F | P | L | R | A | E | W | E | R | Q | P | L | Q | L | R | L | F | G | E | H | L | A | G | E | G | F | F | E | R | L | A | A | L | - | - | R | L | A | P | H | E | N |
| C_violaceum | A | F | C | A | L | M | D | E | I | I | L | S | S | D | - | - | - | - | F | A | L | R | D | E | W | E | R | M | P | L | Q | L | R | L | F | G | E | H | L | A | G | E | G | F | F | N | R | L | E | Q | L | - | - | R | N | H | P | A | E | N |
| P_putida | A | L | C | A | F | L | D | E | S | V | L | R | S | D | D | - | - | - | N | A | L | R | R | H | F | E | L | Q | P | L | Q | F | R | Y | F | G | V | H | L | A | G | E | G | F | F | E | K | V | D | A | L | - | - | R | A | D | V | K | Q | N |
| C_Solibacter | A | T | V | A | F | L | D | E | S | V | L | N | S | G | - | - | - | - | N | P | I | F | S | E | W | L | R | K | P | L | Q | A | E | L | F | G | T | H | T | A | G | E | E | F | F | V | S | L | Q | Q | L | L | G | R | A | D | S | A | D | L |
| E_tarda | A | V | V | A | W | M | D | E | T | I | M | C | S | T | - | - | - | - | W | D | G | V | A | T | W | R | R | D | P | L | Q | A | S | Y | F | N | T | V | C | A | G | V | D | F | F | D | K | L | T | A | L | - | - | - | - | - | S | P | E | A |
| P_aeruginosa_HSI-III | A | F | V | A | L | V | D | E | T | L | V | F | S | A | - | - | - | - | W | P | G | Q | G | A | W | Q | D | K | P | L | E | S | H | L | Y | G | S | R | Q | A | G | E | Y | L | P | L | A | I | K | R | L | L | - | D | E | R | A | P | A | S |
| L_pneumophila | L | L | S | A | T | I | D | E | I | L | G | K | S | Y | L | - | - | - | R | V | Y | N | L | T | T | E | F | K | S | F | T | P | L | S | S | D | G | A | Q | P | Q | Q | R | F | F | E | I | L | N | Y | I | - | - | K | E | R | P | N | Q | F |
| D_thiodismutans | P | L | V | A | H | I | D | E | Q | V | Q | F | R | F | - | - | - | - | L | N | P | A | Q | S | N | G | W | P | P | L | Q | R | E | L | F | D | T | D | S | A | G | E | L | F | Y | E | T | L | D | D | L | - | - | L | I | K | P | Q | T | L |
| F_tularensis | P | L | L | A | Y | V | D | E | K | L | M | L | L | R | - | - | - | - | E | K | S | A | S | N | I | S | W | S | L | L | Q | L | E | Y | Y | D | R | K | D | G | G | E | Y | V | F | E | I | T | D | N | I | - | - | L | S | E | N | I | Y | P |
|  |  |  |  |  |  |  |  |  |  |  |  |  |  |  |  |  |  |  |  |  |  |  |  |  |  |  |  |  |  |  |  |  |  |  |  |  |  |  |  |  |  |  |  |  |  |  |  |  |  |  |  |  |  |  |  |  |  |  |  |  |
| V_cholerae | Q | A | L | L | A | F | I | Y | H | C | L | I | L | G | F | E | G | K | Y | R | V | M | E | G | G | Q | A | E | R | E | K | V | I | S | R | L | H | Q | L | L | S | S | L | E | E | S | E | P | Q | D | L | T | R | P | T | D | H | V | V | R |
| Marinomonas | K | D | L | L | E | F | I | Y | Y | C | L | V | L | G | F | E | G | K | F | K | V | L | N | N | G | K | E | E | R | E | K | V | I | S | K | L | H | S | L | L | D | S | M | D | E | K | E | S | L | K | L | T | S | K | N | D | H | V | V | N |
| S_woodyl | R | E | L | L | E | F | I | Y | L | C | L | C | L | G | F | E | G | R | Y | K | V | V | V | N | G | R | E | E | F | D | K | I | V | N | R | L | Y | E | T | L | R | H | L | R | D | E | E | P | E | L | L | S | N | A | T | D | H | V | V | N |
| E_coli | Q | D | L | L | E | F | L | W | L | C | F | S | L | G | F | R | G | R | Y | K | V | A | V | Q | D | Q | G | E | F | E | Q | I | Y | R | R | L | Y | H | V | L | H | K | L | R | G | D | A | P | F | P | L | L | H | Q | D | K | K | T | Q | G |
| S_sonnei | Q | D | L | L | E | F | L | W | I | C | F | S | L | G | F | R | G | R | Y | K | V | A | A | Q | D | Q | G | E | F | E | Q | I | Y | R | R | L | Y | H | V | L | H | K | L | R | G | D | A | P | F | P | L | L | H | Q | D | K | K | T | Q | G |
| Y_pestis | Q | D | L | L | E | F | I | Y | L | C | F | C | L | G | Y | R | G | R | Y | K | V | T | S | Q | N | G | D | D | F | E | R | L | F | R | R | L | H | H | Q | L | Q | Q | L | R | G | D | A | P | P | T | V | L | Y | Q | G | G | G | R | L | N |
| P_asymbiotica | Q | H | L | L | E | F | I | Y | L | C | F | C | L | G | Y | R | G | R | Y | K | V | S | S | Q | K | G | D | D | F | E | R | L | F | R | R | I | H | Q | Q | L | H | A | L | R | G | E | A | P | P | T | I | L | H | S | N | A | N | E | Q | D |
| P_mirabilis | L | D | L | I | E | F | I | Y | L | C | L | C | L | G | Y | R | G | R | Y | K | V | S | A | G | Q | N | D | E | F | N | H | L | L | R | R | L | Q | K | Q | I | Q | Q | L | R | G | N | A | K | P | I | V | L | F | E | K | G | D | N | Q | Q |
| A_hydrophila | R | D | I | L | E | F | I | Y | L | C | L | C | L | G | F | E | G | R | Y | K | V | M | S | K | G | Q | G | E | F | E | R | I | V | R | Q | L | H | K | Q | L | A | P | E | A | G | G | E | A | P | S | V | F | H | L | D | L | G | Q | Q | A |
| P_wasabiae | R | D | I | L | E | F | I | Y | L | C | L | C | L | G | F | E | G | R | Y | R | V | M | T | Q | G | R | E | E | L | D | R | V | V | S | K | L | H | D | T | L | R | P | E | P | T | - | N | A | P | T | V | F | H | L | N | L | G | Q | Q | A |
| P_aeruginosa_HSI-II | L | P | M | L | E | L | M | Y | L | C | L | A | L | G | F | E | G | K | Y | R | V | M | A | R | G | V | L | D | L | D | G | I | S | D | A | L | Y | R | Q | I | R | Q | L | R | G | D | V | P | R | E | L | S | P | H | W | Q | G | L | S | D |
| R_solanacearum | L | Q | A | L | E | V | F | H | M | C | L | L | L | G | F | R | G | K | Y | I | L | - | - | E | G | P | E | K | L | A | Y | L | T | A | R | L | G | D | E | I | S | A | I | K | G | - | K | R | A | A | F | A | P | H | W | L | L | P | D | K |
| C_taiwanensis | L | Q | A | L | E | V | F | H | M | C | L | L | L | G | F | R | G | K | Y | I | L | - | - | E | G | P | E | K | L | A | Y | L | T | A | R | L | G | D | E | I | S | A | I | K | G | - | K | R | A | A | F | A | P | Q | W | P | I | P | D | K |
| B_pseudomallei | L | Q | S | L | E | V | F | H | M | C | L | L | L | G | F | Q | G | K | Y | L | L | - | - | E | G | P | E | K | L | A | Y | L | T | A | R | L | G | D | E | I | A | N | M | K | G | - | K | R | A | P | F | A | P | H | W | P | L | P | D | Q |
| D_aromatica | I | N | A | L | E | V | F | H | M | C | L | L | I | G | F | K | G | R | Y | L | L | - | - | E | G | P | E | K | L | K | Y | L | T | L | Q | L | G | E | Q | I | A | H | I | K | G | - | K | A | A | T | F | A | P | N | W | A | A | P | D | T |
| X_oryzae | L | P | S | L | E | I | Y | H | Y | C | L | L | L | G | F | E | G | K | Y | R | L | - | - | E | G | P | E | K | L | G | Y | L | T | A | R | L | G | D | E | I | V | Y | F | K | G | - | K | R | S | G | F | A | P | H | W | P | P | P | D | N |
| Acinetobacter | L | T | S | L | E | V | F | H | Y | C | L | L | L | G | F | Q | G | K | Y | R | I | - | - | E | S | I | E | S | L | N | H | L | V | A | R | V | G | D | E | I | D | Y | L | K | G | - | K | K | A | A | F | S | P | F | S | A | I | P | D | Q |
| Azoarcus | I | E | C | L | E | V | F | H | A | C | L | L | L | G | F | R | G | K | Y | L | L | - | - | D | D | S | D | R | I | R | Y | L | Q | R | T | L | A | Q | E | L | Q | R | V | R | G | - | D | P | Q | T | P | P | A | L | W | K | L | P | E | E |
| C_violaceum | I | E | P | L | E | V | F | Y | T | C | L | L | L | G | F | Q | G | K | Y | L | L | - | - | E | G | Q | E | K | L | G | Y | L | T | H | K | L | G | Q | E | I | Q | Q | V | R | G | - | G | K | A | D | F | A | P | N | W | Q | L | P | Q | R |
| P_putida | I | D | V | L | E | V | Y | H | L | C | L | A | L | G | F | E | G | K | F | S | L | - | - | G | Q | K | D | Q | L | R | Y | L | A | N | T | L | G | Q | D | I | A | R | Y | R | K | - | A | P | K | A | L | S | P | D | W | A | L | P | D | Q |
| C_Solibacter | A | D | L | I | E | I | H | Y | L | C | L | L | L | G | F | G | G | R | Y | S | A | - | - | G | N | R | G | E | L | A | Q | I | M | N | L | T | G | E | K | I | R | R | I | R | G | R | F | G | A | L | S | P | S | W | Q | P | S | N | E | T |
| E_tarda | D | D | V | R | E | V | Y | L | L | C | L | A | L | G | F | E | G | R | Y | A | G | P | T | G | Q | T | A | L | T | Q | I | R | V | H | Q | L | R | D | L | H | G | E | V | W | E | A | S | R | R | L | F | P | E | A | Y | P | R | S | S | V |
| P_aeruginosa_HSI-III | R | D | L | A | N | V | Y | L | Q | C | L | I | L | G | F | R | G | R | L | R | G | P | R | G | E | A | L | H | E | K | W | R | Q | A | L | F | A | F | A | W | Q | R | E | A | D | A | A | D | L | G | R | R | L | E | Q | P | A | A | A | P |
| L_pneumophila | L | D | L | I | E | L | A | Y | F | C | L | I | A | G | F | E | G | E | Y | H | L | K | A | D | G | R | Q | C | L | D | N | T | I | E | D | L | Y | Q | I | I | Q | K | Y | R | F | N | K | P | H | R | L | F | N | E | N | P | L | P | K | T |
| D_thiodismutans | P | L | I | L | E | V | Y | Y | Y | C | L | N | E | G | F | G | G | R | L | A | N | - | - | - | N | P | S | K | R | Q | E | Y | M | E | R | L | R | N | R | I | P | T | P | S | Q | - | - | Q | D | E | T | V | P | F | P | V | E | E | V | Q |
| F_tularensis | E | I | C | Y | Q | T | I | S | L | I | L | H | N | D | F | Y | G | K | Y | Y | D | N | I | Y | N | H | S | F | L | A | Y | K | K | E | I | D | K | H | I | E | N | S | T | I | D | S | V | N | F | I | D | I | P | V | N | S | P | P | L | S |
|  |  |  |  |  |  |  |  |  |  |  |  |  |  |  |  |  |  |  |  |  |  |  |  |  |  |  |  |  |  |  |  |  |  |  |  |  |  |  |  |  |  |  |  |  |  |  |  |  |  |  |  |  |  |  |  |  |  |  |  |  |
| V_cholerae | A | K | Y | T | L | S | R | Q | M | P | V | W | - | S | V | F | A | G | F | I | V | L | W | V | G | L | F | L | G | Y | S | Y | V | L | H | S | K | S | S | D | V | L | N | Q | L | N | Q | I | L | - | - | - | - | - | - | - | - | - | - | - |
| Marinomonas | T | R | Y | K | L | S | K | Q | L | P | V | W | - | S | V | F | A | F | F | A | A | F | W | G | L | T | F | C | G | Y | L | F | L | L | N | S | K | S | S | N | V | L | M | Q | L | N | Q | I | L | Q | - | - | - | - | - | - | - | - | - | - |
| S_woodyl | T | K | F | Q | I | G | R | Q | M | P | I | W | - | T | I | F | A | G | F | F | V | M | L | A | V | I | F | I | F | Y | S | V | S | L | A | N | K | S | A | G | V | L | D | Q | L | F | Q | I | L | N | - | - | - | - | - | - | - | - | - | - |
| E_coli | G | R | Y | Q | L | I | S | R | L | T | V | K | - | H | I | F | C | G | G | V | V | V | L | A | L | F | Y | L | F | Y | L | L | R | L | D | S | Q | T | Q | D | I | L | H | Q | L | N | K | L | L | R | - | - | - | - | - | - | - | - | - | - |
| S_sonnei | G | R | Y | Q | L | I | S | R | L | T | V | K | - | H | I | F | C | G | G | V | V | V | L | A | L | F | Y | L | F | Y | L | L | R | L | D | S | Q | T | Q | D | I | L | H | Q | L | N | K | L | L | R | - | - | - | - | - | - | - | - | - | - |
| Y_pestis | S | R | Y | H | L | S | K | R | L | T | I | K | - | H | L | L | W | G | G | V | S | L | L | V | V | I | Y | L | F | Y | A | I | H | L | H | T | Q | S | Q | D | I | L | Q | Q | L | N | N | L | L | S | - | - | - | - | - | - | - | - | - | - |
| P_asymbiotica | S | R | Y | R | L | G | K | R | L | T | I | K | - | H | L | F | W | S | G | I | G | L | L | A | V | V | Y | G | F | Y | A | V | R | L | H | N | Q | T | Q | N | I | L | Q | Q | L | N | N | L | L | S | - | - | - | - | - | - | - | - | - | - |
| P_mirabilis | S | R | Y | R | L | G | K | R | L | G | A | R | - | Y | I | L | I | G | T | A | V | F | A | L | I | I | Y | S | I | Y | S | S | R | L | N | Y | Q | T | L | S | I | V | E | Q | L | N | T | L | L | G | - | - | - | - | - | - | - | - | - | - |
| A_hydrophila | S | R | Y | Q | L | R | K | Q | V | S | L | R | - | S | L | F | M | G | G | A | L | I | L | A | L | I | F | G | L | Y | H | H | Q | L | N | N | Q | T | Q | D | V | L | R | Q | L | G | E | L | L | K | - | - | - | - | - | - | - | - | - | - |
| P_wasabiae | S | R | Y | Q | L | R | R | Q | V | S | L | R | - | T | L | F | I | G | V | C | V | A | L | V | A | A | F | G | L | Y | R | Y | Q | L | T | H | Q | T | Q | D | V | L | R | Q | L | G | E | L | L | Q | - | - | - | - | - | - | - | - | - | - |
| P_aeruginosa_HSI-II | Q | R | R | G | L | V | R | I | V | P | W | W | - | M | V | A | L | F | T | L | V | C | L | V | V | M | Y | S | G | F | A | W | V | L | G | E | Q | R | D | T | V | L | Q | P | Y | Q | S | L | D | A | A | A | G | Q | P | G | S | - | - | - |
| R_solanacearum | V | S | H | R | L | K | R | E | T | P | L | W | - | I | F | G | A | V | F | A | L | I | A | L | L | G | Y | I | G | L | S | S | T | L | R | A | K | T | N | E | T | L | E | G | Y | S | Q | V | I | K | L | G | P | R | F | S | H | L | T | I |
| C_taiwanensis | I | S | H | A | L | K | R | E | T | P | L | W | - | I | F | G | A | V | F | A | L | I | A | L | L | A | F | L | G | L | S | T | T | L | R | S | Q | T | S | D | T | L | Q | G | Y | S | Q | V | I | K | L | G | P | R | Y | S | H | L | T | I |
| B_pseudomallei | I | S | H | R | L | K | R | E | V | P | L | W | - | A | I | G | A | V | F | A | L | V | G | L | L | A | F | V | G | L | N | T | Y | L | K | D | S | T | V | R | A | L | A | P | Y | S | Q | V | I | K | V | G | P | E | S | A | N | L | T | I |
| D_aromatica | I | S | N | A | I | K | R | D | I | P | F | W | - | V | I | T | S | V | L | A | L | L | G | L | V | A | Y | I | G | L | D | W | H | A | G | S | T | V | Q | N | T | L | S | P | F | K | N | I | V | Q | L | A | P | R | A | P | T | L | T | I |
| X_oryzae | V | R | H | A | L | R | R | V | V | P | L | W | - | L | P | A | A | L | V | T | G | F | G | L | L | G | F | F | G | L | R | L | S | L | D | H | Q | T | E | R | R | M | A | A | Y | N | Q | V | V | Q | M | P | Q | R | T | A | H | I | T | I |
| Acinetobacter | I | R | H | M | I | H | G | E | L | P | F | F | - | W | I | L | I | I | L | L | I | F | A | L | L | T | F | G | G | L | R | Y | M | L | S | H | Q | T | D | R | A | L | A | P | Y | Q | N | V | V | S | A | P | A | E | E | A | H | I | T | I |
| Azoarcus | E | L | T | P | P | R | P | G | L | P | T | R | - | F | Y | A | G | I | L | L | V | V | G | L | A | F | F | A | L | Y | Q | V | L | L | D | Q | R | A | Q | A | L | F | G | V | - | - | - | - | - | - | - | - | - | - | - | - | - | - | - | - |
| C_violaceum | F | Q | A | F | V | R | H | E | L | P | L | W | - | L | Y | F | A | L | L | A | I | V | G | A | G | I | F | V | A | F | R | W | L | L | A | R | Q | A | A | S | A | F | G | L | - | - | - | - | - | - | - | - | - | - | - | - | - | - | - | - |
| P_putida | V | S | Q | M | L | R | H | E | V | P | L | W | - | V | Y | L | A | L | I | A | L | V | C | V | A | V | Y | L | T | L | D | W | L | L | D | K | D | V | A | A | L | S | E | Q | I | R | Q | L | F | S | A | - | - | - | - | - | - | - | - | - |
| C_Solibacter | V | A | S | Q | S | D | P | W | V | K | R | W | G | I | V | A | A | V | C | A | L | V | T | V | L | L | F | A | G | Y | M | F | G | L | S | S | V | V | S | Q | L | R | T | L | S | T | Q | G | K | G | - | - | - | - | - | - | - | - | - | - |
| E_tarda | T | P | T | Q | Q | T | R | W | L | T | Q | S | R | F | T | T | V | V | V | P | L | V | I | L | V | A | L | Y | G | I | L | N | W | V | L | Y | E | Y | A | D | L | A | L | E | W | L | P | - | - | - | - | - | - | - | - | - | - | - | - | - |
| P_aeruginosa_HSI-III | P | R | R | L | P | V | R | A | A | L | P | D | G | F | R | L | G | L | A | V | L | G | L | V | L | L | M | S | G | I | G | H | L | F | W | R | D | I | Q | H | E | M | A | A | V | T | H | L | A | D | A | E | Q | A | P | - | - | - | - | - |
| L_pneumophila | I | K | K | N | Y | K | A | T | W | I | T | T | - | - | - | - | I | A | A | V | G | I | V | I | L | A | F | V | T | S | H | V | L | L | E | N | K | A | K | T | V | L | F | G | H | T | Q | L | A | M | L | E | N | - | - | - | - | - | - | - |
| D_thiodismutans | S | S | S | M | L | S | T | I | S | P | F | W | - | - | F | Y | C | S | A | A | V | A | T | G | L | V | Y | A | G | L | K | L | L | G | H | Y | W | T | P | F | - | - | - | - | - | - | - | - | - | - | - | - | - | - | - | - | - | - | - | - |
| F_tularensis | R | K | Y | S | K | T | L | K | F | L | L | R | I | G | V | P | L | G | L | F | L | L | S | L | L | I | L | L | S | W | - | - | - | - | - | - | - | - | - | - | - | - | - | - | - | - | - | - | - | - | - | - | - | - | - | - | - | - | - | - |
|  |  |  |  |  |  |  |  |  |  |  |  |  |  |  |  |  |  |  |  |  |  |  |  |  |  |  |  |  |  |  |  |  |  |  |  |  |  |  |  |  |  |  |  |  |  |  |  |  |  |  |  |  |  |  |  |  |  |  |  |  |
