# Supplementary material for: DotU and VgrG, Core Components of Type VI Secretion Systems, Are Essential for Francisella LVS Pathogenicity
Source: PLoS One. 2012 Apr 13;7(4):e34639. doi: 10.1371/journal.pone.0034639 (PMC3326028; doi:10.1371/journal.pone.0034639)
Supplement: Table S1 — Strains and plasmids used in this study. (DOCX) [file pone.0034639.s003.docx]

Table S1. Strains and plasmids used in this study

| Strain or plasmid | Relevant genotype or phenotype | Source or reference |
| --- | --- | --- |
| Strain |  |  |
| *E. coli* |  |  |
| TOP10 | F-*mcrA*, Δ(*mrr*-*hsdRMS-mcrBC*), φ80*lacZ*ΔM15, Δ*lacX*74, *recA1*, *deoR*, *araD*139, Δ(*ara-leu*)7679, *galU*, *galK*, *rpsL* (Str^R^), *endA1*, *nupG* | Invitrogen |
| S17-1λ*pir* | *recA*, *thi*, *pro*, *hsdR^-^M^+^*, Sm^R^, <RP4:2-Tc:Mu:Ku:Tn7>Tp^R^ | [[73](#_ENREF_73)] |
| DH5αF′IQ | F-φ80*lac*ZΔM15 Δ(*lac*ZYA-*arg*F) U169 *rec*A1 *end*A1 *hsd*R17 (rk-, mk+) *pho*A *sup*E44 λ- *thi*-1 *gyr*A96 *rel*A1/F´ *pro*AB+ *lac*IqZΔM15 zzf::Tn5 [Km^R^]. | Invitrogen |
| KDZif1ΔZ | B2H reporter strain, Km^R^, Cml^R^ | [[74](#_ENREF_74)] |
| *F. tularensis* |  |  |
| LVS | Live vaccine strain | USAMRIID^1^ |
| Δ*iglA* | LVS, *iglA* in-frame deletion of codons 4-174 | [[14](#_ENREF_14)] |
| Δ*iglB* | LVS, *iglB* in-frame deletion of codons 54-346 | [[14](#_ENREF_14)] |
| Δ*iglC* | LVS, *iglC* in-frame deletion of codons 28-205 | [[75](#_ENREF_75)] |
| Δ*iglD* | LVS, *iglD* in-frame deletion mutant | [[68](#_ENREF_68)] |
| Δ*dotU* | LVS, *dotU* in-frame deletion of codons 4-203 | This study |
| Δ*vgrG* | LVS, *vgrG* in-frame deletion of codons 4-162 | This study |
| Δ*vgrG*/VgrGcis | Δ*vgrG*, in *cis* complementation of one *vgrG* deletion copy | This study |
| *S. cerevisiae* |  |  |
| AH109 | *MATα*, *trp1-901*, *leu2-3*, *112*, *ura3-52*, *his3-200*, *gal4Δ*, *gal80Δ*, *LYS2::GAL1_UAS_-GAL1_TATA_-HIS3*, *GAL2_UAS_GAL2_TATA_-ADE2*, *URA3::MEL1_UAS_-MEL1_TATA_-lacZ*, *MEL1* | Clontech Laboratories |
| Plasmid |  |  |
| pCR^®^4-TOPO^®^ | TA cloning vector, Km^R^, Cb^R^ | Invitrogen |
| pDM4 | Suicide plasmid carrying *sacBR*, Cm^R^ | [[76](#_ENREF_76)] |
| pMOL72 | pDM4 carrying a *Xho*I/*Sac*I PCR fragment of Δ*vgrG*_4-162_ with flanking regions, Cm^R^ | This study |
| pJEB752 | pDM4 carrying a *Xho*I/*Sac*I PCR fragment of Δ*dotU*_4-203_ with flanking regions, Cm^R^ | This study |
| pJEB926 | pDM4 carrying a *Xho*I/*Sac*I PCR fragment of *vgrG* with flanking regions, Cm^R^ | This study |
| pKK289Km | expression plasmid carrying a *gfp* gene under the control of the LVS *groE* promoter, Km^R^ | [[68](#_ENREF_68)] |
| pMOL128 | pKK289Km encoding DotU, Km^R^ | This study |
| pMOL71 | pKK289Km encoding VgrG, Km^R^ | This study |
| pJEB390 | pKK289Km encoding IglA-GSK, Km^R^ | This study |
| pJEB391 | pKK289Km encoding IglC-GSK, Km^R^ | This study |
| pMOL52 | pKK289Km derivative used to construct C-terminal fusion proteins to eukaryotic GSK, Km^R^ | [[23](#_ENREF_23)] |
| pMOL58 | pMOL52 encoding DotU-GSK, Km^R^ | This study |
| pJEB914 | pMOL52 encoding DotU _DE70-71AA_-GSK, Km^R^ | This study |
| pJEB915 | pMOL52 encoding DotU _DE70-71KK_-GSK, Km^R^ | This study |
| pJEB916 | pMOL52 encoding DotU _DE70-71SS_-GSK, Km^R^ | This study |
| pJEB917 | pMOL52 encoding DotU _G134A_-GSK, Km^R^ | This study |
| pJEB918 | pMOL52 encoding DotU _G134S_-GSK, Km^R^ | This study |
| pJEB919 | pMOL52 encoding DotU _G134K_-GSK, Km^R^ | This study |
| pJEB920 | pMOL52 encoding DotU _G134D_-GSK, Km^R^ | This study |
| pMOL54 | pMOL52 encoding VgrG-GSK, Km^R^ | This study |
| pJEB702 | pMOL52 encoding IglB-GSK, Km^R^ | This study |
| pJEB703 | pMOL52 encoding IglD-GSK, Km^R^ | This study |
| pACTR-AP-Zif | B2H vector, directs the synthesis of a Zif268-DNA binding domain fusion protein, Tet^R^ | [[74](#_ENREF_74)] |
| pACTR-MglA-Zif | pACTR-AP-Zif encoding MglA, Tet^R^ | [[61](#_ENREF_61)] |
| pJEB871 | pACTR-AP-Zif encoding DotU, Tet^R^ | This study |
| pJEB873 | pACTR-AP-Zif encoding VgrG, Tet^R^ | This study |
| pJEB876 | pACTR-AP-Zif encoding IcmF, Tet^R^ | This study |
| pMOL135 | pACTR-AP-Zif encoding IglA, Tet^R^ | This study |
| pMOL139 | pACTR-AP-Zif encoding IglB, Tet^R^ | This study |
| pLM5 | pACTR-AP-Zif encoding IglC, Tet^R^ | This study |
| pBRGPω | B2H vector, directs the synthesis of a Gal11P-ω fusion protein, Cb^R^ | [[74](#_ENREF_74)] |
| pBRSspA-ω | pBRGPω encoding SspA, Cb^R^ | [[61](#_ENREF_61)] |
| pJEB872 | pBRGPω encoding DotU, Cb^R^ | This study |
| pJEB874 | pBRGPω encoding VgrG, Cb^R^ | This study |
| pJEB877 | pBRGPω encoding IcmF, Cb^R^ | This study |
| pMOL133 | pBRGPω encoding IglA, Cb^R^ | This study |
| pMOL134 | pBRGPω encoding IglB, Cb^R^ | This study |
| pLM6 | pBRGPω encoding IglC, Cb^R^ | This study |
| pGADT7 | Y2H vector, *LEU2*, Cb^R^ | Clontech Laboratories |
| pMOL47 | pGADT7 encoding DotU, *LEU2*, Cb^R^ | This study |
| pMOL31 | pGADT7 encoding VgrG, *LEU2*, Cb^R^ | This study |
| pJEB545 | pGADT7 encoding IcmF_363-1093_, *LEU2*, Cb^R^ | [[14](#_ENREF_14)] |
| pJEB890 | pGADT7 encoding IcmF, *LEU2*, Cb^R^ | This study |
| pJEB393 | pGADT7 encoding IglA, *LEU2*, Cb^R^ | [[14](#_ENREF_14)] |
| pJEB395 | pGADT7 encoding IglB, *LEU2*, Cb^R^ | [[14](#_ENREF_14)] |
| pJEB397 | pGADT7 encoding IglC, *LEU2*, Cb^R^ | [[14](#_ENREF_14)] |
| pGBKT7 | Y2H vector, *TRP1*, Km^R^ | Clontech Laboratories |
| pMOL51 | pGBKT7 encoding DotU, *TRP1*, Km^R^ | This study |
| pMOL37 | pGBKT7 encoding VgrG, *TRP1*, Km^R^ | This study |
| pJEB546 | pGBKT7 encoding IcmF_363-1093_, *TRP1*, Km^R^ | [[14](#_ENREF_14)] |
| pJEB891 | pGBKT7 encoding IcmF, *TRP1*, Km^R^ | This study |
| pJEB392 | pGBKT7 encoding IglA, *TRP1*, Km^R^ | [[14](#_ENREF_14)] |
| pJEB394 | pGBKT7 encoding IglB, *TRP1*, Km^R^ | [[14](#_ENREF_14)] |
| pJEB396 | pGBKT7 encoding IglC, *TRP1*, Km^R^ | [[14](#_ENREF_14)] |

^1^ US Army Medical Research Institute of Infectious Diseases, Fort Detrick, Frederick, MD.
