# Supplementary material for: DotU and VgrG, Core Components of Type VI Secretion Systems, Are Essential for Francisella LVS Pathogenicity
Source: PLoS One. 2012 Apr 13;7(4):e34639. doi: 10.1371/journal.pone.0034639 (PMC3326028; doi:10.1371/journal.pone.0034639)
Supplement: Table S2 — Oligonucleotides used in this study. (DOCX) [file pone.0034639.s004.docx]

Table S2. Oligonucleotides used in this study

| Purpose | Oligonucleotide pair(s) |
| --- | --- |
| *LVS null mutants* |  |
| DotU Δ4-203 | DotU_a: 5´-*CTC GAG* GAT ATT ATT GAT GTG TTG CAT ATT-3´ (*Xho*I) and DotU_b: 5´-*GGA TCC* TTA TTA AGC TGG TAA TAA GGG TA-3´ (*Bam*HI)  DotU_c: 5´-*GGA TCC* GTC TTT CAT TTA TAA TAT CCT TTA T-3´ (*Bam*HI), DotU_d: 5´-*GAG CTC* ATA GAA GAC AAG AGA ACA GGT A-3´ (*Sac*I) |
| VgrG Δ4-162 | Del_pigB_a: 5´-*CTC GAG* TAG TGT TTC TTC ATC AAA CAT TA-3´(*Xho*I) and Del_pigB_b: 5´-ATT CTT ATG TCA AAA GTT GGA TAA TAA TAT GAA TAA T-3´  Del_pigB_c:5´-TTT TGA CAT AAG AAT ATC CCC T-3´ and Del_PigB_d: 5´-*GAG CTC* ATC AAT TCC CTT TTA ATA AGG AT-3´(*Sac*I) |
| *Complementation in* cis |  |
| VgrG | Del_pigB_a (*Xho*I) and Del_PigB_d (*Sac*I) |
| *Complementation in* trans |  |
| DotU | PigF_F: 5´-*ATT AAT* GAA AGA CTT TAA AGA GAT AGA-3´ (*Ase*I) and PigF_R: 5´-*GAA TTC* TTA CCA GCT TAA TAA AAT TAG TAA-3´ (*Eco*RI) |
| DotU-GSK | PigF_F (*Ase*I) and PigF_GSKrev: 5´-*GGT ACC* CCA GCT TAA TAA AAT TAG TAA G-3´ (*Kpn*I) |
| DotU _DE70-71AA_-GSK | DotU_NdeI_F :5´-*CAT ATG* AAA GAC TTT AAA GAG ATA GAA ATT-3´ (*Nde*I) and DotU_7071AA_b: 5´-**G**CA **G**CA ACA TAA GCA AGT AAC GGA AAA AC-3´  DotU_7071AA_c: 5´-TTG CTT ATG TTG **C**TG **C**AA AAT TGA TGC TAC TTA GGG AA-3´ and PigF_GSKrev (*Kpn*I) |
| DotU _DE70-71KK_-GSK | DotU_NdeI_F (*Nde*I) and DotU_7071KK_b: 5´-**TC**T **T**AA CAT AAG CAA GTA ACG GAA AAA C-3´  DotU_7071KK_c: 5´-CTT GCT TAT GTT **A**A**G A**AA AAA TTG ATG CTA CTT AGG GA-3´ and PigF_GSKrev (*Kpn*I) |
| DotU _DE70-71SS_-GSK | DotU_NdeI_F (*Nde*I) and DotU_7071SS_b: 5´-**GA**A **GA**A ACA TAA GCA AGT AAC GGA AAA AC-3´  DotU_1771SS_c: 5´-TTG CTT ATG TT**T C**T**T** **C**AA AAT TGA TGC TAC TTA GGG AA-3´ and PigF_GSKrev (*Kpn*I) |
| DotU _G134A_-GSK | DotU_NdeI_F (*Nde*I) and DotU_134A_b: 5´-G**G**C ATA GAA GTC ATT GTG TAG GAT A-3´  DotU_134A_c: 5´- CAC AAT GAC TTC TAT G**C**C AAA TAC TAT GAC AAT ATA TAT AAC-3´ and PigF_GSKrev (*Kpn*I) |
| DotU _G134S_-GSK | DotU_NdeI_F (*Nde*I) and DotU_134S_b: 5´-**T**AT AGA AGT CAT TGT GTA GGA TA-3´  DotU_134S_c: 5´-CAC AAT GAC TTC TAT **A**GC AAA TAC TAT GAC AAT ATA TAT AAC-3´ and PigF_GSKrev (*Kpn*I) |
| DotU _G134K_-GSK | DotU_NdeI_F (*Nde*I) and DotU_134K_b: 5´-**TTT** ATA GAA GTC ATT GTG TAG GAT A-3´  DotU_134K_c: 5´-CAA TGA CTT CTA T**AA A**AA ATA CTA TGA CAA TAT ATA TAA C-3´ and PigF_GSKrev (*Kpn*I) |
| DotU _G134D_-GSK | DotU_NdeI_F (*Nde*I) and DotU_134D_b: 5´- G**T**C ATA GAA GTC ATT GTG TAG GAT A-3´  DotU_134D_c: 5´-CAC AAT GAC TTC TAT G**A**C AAA TAC TAT GAC AAT ATA TAT AAC-3´ and PigF_GSKrev (*Kpn*I) |
| VgrG | PigB_F: 5´-*CAT ATG* TCA AAA GCA GAC CAT ATT T-3´ (*Nde*I) and PigB_R: 5´-*GAA TTC* TTA TCC AAC CAT TGT TGC TGT AG-3´ (*Eco*RI) |
| VgrG-GSK | PigB_F (*Nde*I) and PigB_GSKrev: 5´-*GGT ACC* TCC AAC CAT TGT TGC TGT AG- 3´ (*Kpn*I) |
| IglA-GSK | IglA_GSK_F: 5´-*CAT ATG* GCA AAA AAT AAA ATC CCA AAT TCA AGG-3´ (*Nde*I) and IglA_GSK_R: 5´-TAT GAT TCA GCA AAT GAA GTA GTT CTT GGT CTA CCT GAC ATC TTA CCA TCT ACT TGT TGA TTA CTT AAG TC-3´ |
| IglB-GSK | IglB_Y2H_F: 5´-*CAT ATG* ACA ATA AAT AAA TTA AGT CTC ACT GAT G-3´ (*Nde*I) and IglB_KpnI_R: 5´-*GGT ACC* GTT ATT ATT TGT ACC GAA TAA TTC-3´ (*Kpn*I) |
| IglC-GSK | IglC_GSK_F: 5´-*CAT ATG* AGT GAG ATG ATA ACA AGA CAA CAG GTA-3´ (*Nde*I) and IglC_GSK_R: 5´-TAT GAT TCA GCA AAT GAA GTA GTT CTT GGT CTA CCT GAC ATT GCA GCT GCA ATA TAT CCT ATT TTA GCA-3´ |
| IglD-GSK | IglD_Y2H_F: 5´-*CAT ATG* TTT CTA GAA AGG ATT TAT TGG GAA GAT-3´(*Nde*I) and IglD_KpnI_R: 5´-*GGT ACC* AGA AAA GGC TAT AAA GAA ATC AA-3´ (*Kpn*I) |
| *Yeast two-hybrid interaction studies* |  |
| DotU | PigF_F (*Nde*I) and PigF_R (*Eco*RI) |
| VgrG | PigB_F (*Nde*I) and PigB_R (*Eco*RI) |
| IcmF | PdpB_NdeI_F: 5´-*CAT ATG* AAT TTT ATT AAA AAT CAT CAA ATA TT-3´ (*Nde*I) and PdpB_XmaI_R: 5´-*CCC GGG* TTA TTG TAC ATT GAC TTC TCC TTG T-3´ (*Xma*I) |
| *Bacterial two-hybrid interaction studies* |  |
| DotU | DotU_NdeI_F (NdeI) and DotU_mut_b: ´-**A**GC AAG TAA CGG AAA AAC TAT ATA TT-3´  DotU_mut_c: 5´-TTT CCG TTA CTT GC**T** TAT GTT GAT GAA AAA TTG ATG CTA-3´ and DotU_NotI_R: 5´-*GCG GCC GC* CCA GCT TAA TAA AAT TAG TAA GCT T-3´ (*Not*I) |
| VgrG | VgrG_NdeI_F: 5´-*CAT ATG* TCA AAA GCA GAC CAT ATT TTC AAC-3´ (*Nde*I) and VgrG_NotI_R: 5´-*GCG GCC GC* TCC AAC CAT TGT TGC TGT AGA AC-3´ (*Not*I) |
| IcmF | PdpB_NdeI_F: 5´-*CAT ATG* AAT TTT ATT AAA AAT CAT CAA ATA TT-3´ (*Nde*I) and PdpB_mut_b: 5´- **A**GT ATC ATT ATA TTT TGG TAA GAT TAC-3´  PdpB_mut_c: 5´-AAA ATA TAA TGA TAC **T**TA TGA CTT ATC AAT GAT TAC ATC A-3´ and PdpB_NotI_R: 5´-*GCG GCC GC* TTG TAC ATT GAC TTC TCC TTG T-3´ (*Not*I) |
| IglA | IglA_GSK_F (*Nde*I) and IglA_NotI_R: 5´-*GCG GCC GC* CTT ACC ATC TAC TTG TTG ATT ACT-3´ (*Not*I) |
| IglB | IglB_Y2H_F (*Nde*I) and IglB_NotI_R: 5´-*GCG GCC GC* GTT ATT ATT TGT ACC GAA TAA TTC TG-3´ (*Not*I) |
| IglC | IglC_GSK_F (*Nde*I) and IglC_NotI_rev: *GCG GCC GC* TGC AGC TGC AAT ATA TCC TAT-3´(*Not*I) |
| *qPCR* |  |
| *icmF* | FTT1345-F: 5´-TTG AGT CTA AGA GTT ATG CG ACT-3´ and FTT1345-R: 5´-TGT AGT GGT TCA TAT CCT TGT TTG-3´ |
| *dotU* | FTT1351-F: 5´-CTA CAC AAT GAC TTC TAT GGC AAA-3´ and FTT1351-R: 5´-ATT AAC CGA ATC AAT TGT CGA AT-3´ |
| *vgrG* | FTT1347-F: 5´-TTG TTA GCT ACT AAG AAA TCA AGC ATT and FTT1347-R:5´-TTA ATA GTT GCA GAC TCT AGC GAA A-3´ |
| *tul4* | 17kD-F: 5´-GTG CCA TGA TAC AAG CTT CC-3´ and 17kD-R: 5´-GCT GTC CAC TTA CCG CTT CA-3´ |

The nucleotide sequences in italics represent the incorporated *Nde*I, *Eco*RI, *Bam*HI, *Sac*I, *Xma*I, *Xho*I, *Not*I, *Ase*I and *Kpn*I restriction sites used for cloning of the PCR amplified DNA fragments. The underlined sequence indicates the complementary overlap between respective primers in the overlap PCR reactions. In primers used to generate amino acid substitutions, the nucleotides substituted are indicated in boldface. To optimise expression, all substitutions were adapted according to the codon usage preferences of *F*. *tularensis* (http://www.kazusa.or.jp/codon).
